# Structural Modeling of NTPDase-Substrate Complexes Preserving Catalytic Experimental Features

**DOI:** 10.1021/acsomega.5c04628

**Published:** 2025-09-22

**Authors:** João Victor B. de Moraes, Marcelo D. Polêto, Raissa B. de Castro, Gustavo C. Bressan, Raphael de S Vasconcellos, Jean Sévigny, Juliana R. Fietto

**Affiliations:** † General Biology Department, Universidade Federal de Viçosa, Viçosa, Minas Gerais 36570 900, Brazil; ‡ Laboratório de Biologia Teórica e Computacional (LBTC), Universidade de Brasília, Brasília, Distrito Federal 70910-900, Brazil; § Department of Biotechnology, Universidade de São Paulo, Lorena, São Paulo 12602-810, Brazil; ∥ Biochemistry and Molecular Biology Department, Universidade Federal de Viçosa, 36570 900 Viçosa, Minas Gerais Brazil; ⊥ Département de Microbiologie-Infectiologie et d’immunologie, Centres PROTEO et ARThrite, Université Laval, Faculté de Médecine, Quebec city, QC G1 V 0A6, Canada; # Axe Maladies Infectieuses et Immunitaires, Centre de Recherche du CHU de Québec − Université Laval, Quebec city, QC G1 V 4G2, Canada; ∇ Instituto de Biotecnologia Aplicada à Agropecuária (Bioagro), Universidade Federal de Viçosa, Viçosa, Minas Gerais 36570 900, Brazil

## Abstract

Members of the ecto-nucleoside triphosphate diphosphohydrolase (E-NTPDase) family play a pivotal role in hydrolyzing nucleoside triphosphates and diphosphates, modulating purinergic and pyrimidinergic signaling pathways. The NTPDases have therapeutic potential; gaining structural insights into NTPDase-substrate complexes would be valuable for optimizing these enzymes for therapeutic applications. However, such insights remain limited, posing challenges for effective optimization. Molecular docking often fails to capture experimentally characterized substrate conformations, leading to biologically irrelevant models. To address this, we developed a computational strategy that preserves experimentally observed substrate features while leveraging the active site’s conservation across NTPDases. Our method identifies a canonical linear-like substrate conformation encompassing the phosphate tail and nucleobase ring conserved across experimental NTPDase structures. This approach enabled the modeling of *Homo sapiens* (Hs) NTPDases (HsNTPDase1–8) complexed with ATP, ADP, GTP, GDP, UTP, and UDP, accurately positioning metal ion cofactor and catalytic water molecules. The resulting models offer a reliable framework for studying enzyme–substrate interactions, paving the way for rational enzyme engineering and therapeutic exploration.

## Graphical abstract

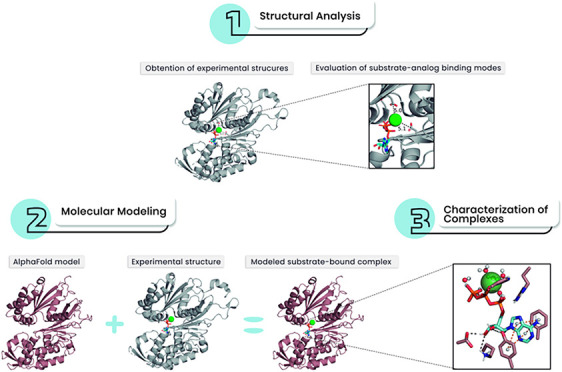

## 1 Introduction

The members of the ecto-nucleoside triphosphate diphosphohydrolase (E-NTPDases or NTPDases) enzyme family play a crucial role in hydrolyzing nucleoside triphosphate (NTP) and diphosphate (NDP) into their monophosphate forms (NMP). Together with ecto-5′-nucleotidase, NTPDases modulate purinergic and pyrimidinergic signaling by modulating the balance of nucleotide forms in the cellular environment.^1–3^ NTPDases have been identified in multiple organisms, where they perform diverse functions, including acting as virulence factors for pathogenic parasites and playing critical roles in immune response and tumor regulation.^4–8^ Although structurally important for biological and therapeutic applications, NTPDase-substrate complexes remain insufficiently characterized, posing challenges for optimizing these enzymes in therapeutic contexts.

To date, 48 NTPDase structures have been experimentally resolved, spanning seven different organisms and defining the general structural features of this enzyme family, including active site residues, substrate binding modes, and catalytic mechanisms.^9–16^ Among these, 15 structures have been resolved in a “productive” substrate-bound state (correctly positioned for catalysis) using purine and pyrimidine substrate analogs.^9,10,12,13^ The notion of what is a “productive” NTPDase-nucleotide complex is based on the set of conserved hydrogen bonds formed between the enzyme and the phosphate moiety of nucleotides, the position of the cofactor, and the presence of six conserved water molecules in the active site for the experimental structures with resolution for the water molecules.^10^ Given the activity of the *Rattus norvegicus* (Rn) NTPDase2 (RnNTPDase2) in crystal form,^9^ this productive binding mode is expected to be a good representation of the precatalytic structure of these complexes. Among the eight human NTPDase isoforms (HsNTPDase1–8), HsNTPDase4 is the only one with an experimentally resolved structure that is currently available. Unfortunately, only the enzyme’s substrate-free (apo) form was captured.^16^

Understanding the NTPDase’s structure and interactions with different substrates can be crucial for rationalizing the enzyme’s specificity, engineering isoforms of interest, and exploring their possible biotechnological applications. In this scenario, computational techniques can offer valuable structural insights into enzyme–substrate complexes and aid studies in understanding the structure/function relationship.^17^ In doing so, enzyme modeling is a critical step that must be carefully considered. Although not replacing experimental structures, AlphaFold2 and 3 enable producing high-quality protein models.^18,19^ In the context of the NTPDases, the protein modeling process can be challenging due to the butterfly-like movement in many isoforms, directly impacting active site structure and its ability to describe a valid substrate-bounded complex.^10,11,16,20^

Another critical step is correctly positioning the substrate into the active site. Molecular docking software is the logical choice for forming an uncharacterized enzyme–substrate complex. Nevertheless, many docking software struggle to adequately describe the binding of nucleotides to target proteins.^21^ Usually, an RMSD of up to 2 Å is used as an efficiency metric for docking experiments.^22,23^ However, a simplistic approach such as this may not be enough when analyzing NTPDase-substrate complexes. As shown in the Supporting Information, imprecisions within this range of variation can lead to unusual substrate conformations, ultimately limiting the biological relevance of the generated complexes (Figure S1). To overcome this limitation, one may base the description of unknown complexes on the available experimental data, thus producing complexes that fit the features observed in experimental structures and do not rely solely on RMSD values.

By carefully examining multiple experimentally resolved NTPDase structures in a productive substrate-bound state with substrate analogs, we developed a robust modeling protocol complemented by MD simulations to accurately position substrates in the active sites of HsNTPDases. This approach preserves key interactions and conserved water molecules, ensuring structural and functional relevance of the generated complexes. Our models provide a more biologically meaningful representation of enzyme–substrate binding, overcoming limitations of standard docking approaches that often fail to capture productive conformations. These refined models offer a reliable framework for investigating substrate specificity, enzyme kinetics, and inhibitor design, addressing critical gaps in NTPDase research. As such, they represent a necessary step forward in understanding and engineering these enzymes for therapeutic and biotechnological applications.

## 2 Results and Discussion

### 2.1 Substrate Interactions within Active Sites in NTPDases are Conserved

The E-NTPDase family members possess a remarkable capability to process diverse nucleotides within a shared active site.^3,24,25^ Given the high degree of conservation of the NTPDase active site in both sequence and structure, it is reasonable to anticipate that the conformation of substrates upon binding would also exhibit some degree of similarity. To evaluate this hypothesis, we made structural comparisons of experimentally resolved substrate analogue structures characterized across various NTPDase isoforms (Table 1) and organisms available in the PDB. As shown in Figure 1, the results support the hypothesis, revealing a consistent substrate conformation upon binding. Among the 15 experimentally resolved structures representing productive analog-bound states available in the PDB, 12 exhibit a shared substrate conformation. This conformation is characterized by the anticonfiguration of the nucleoside, with the phosphate tail and the nucleobase forming a linear-like structure. Figure 1 visually illustrates this substrate conformation (aligned to the *Y*-axis) using the ATP analogs cocrystallized with *R. norvegicus* RnNTPDase2 (A), *Legionella pneumophila* LpNTPDase1 (B), and *Toxoplasma gondii* TgNTPDase3 (C).

**Figure 1:**
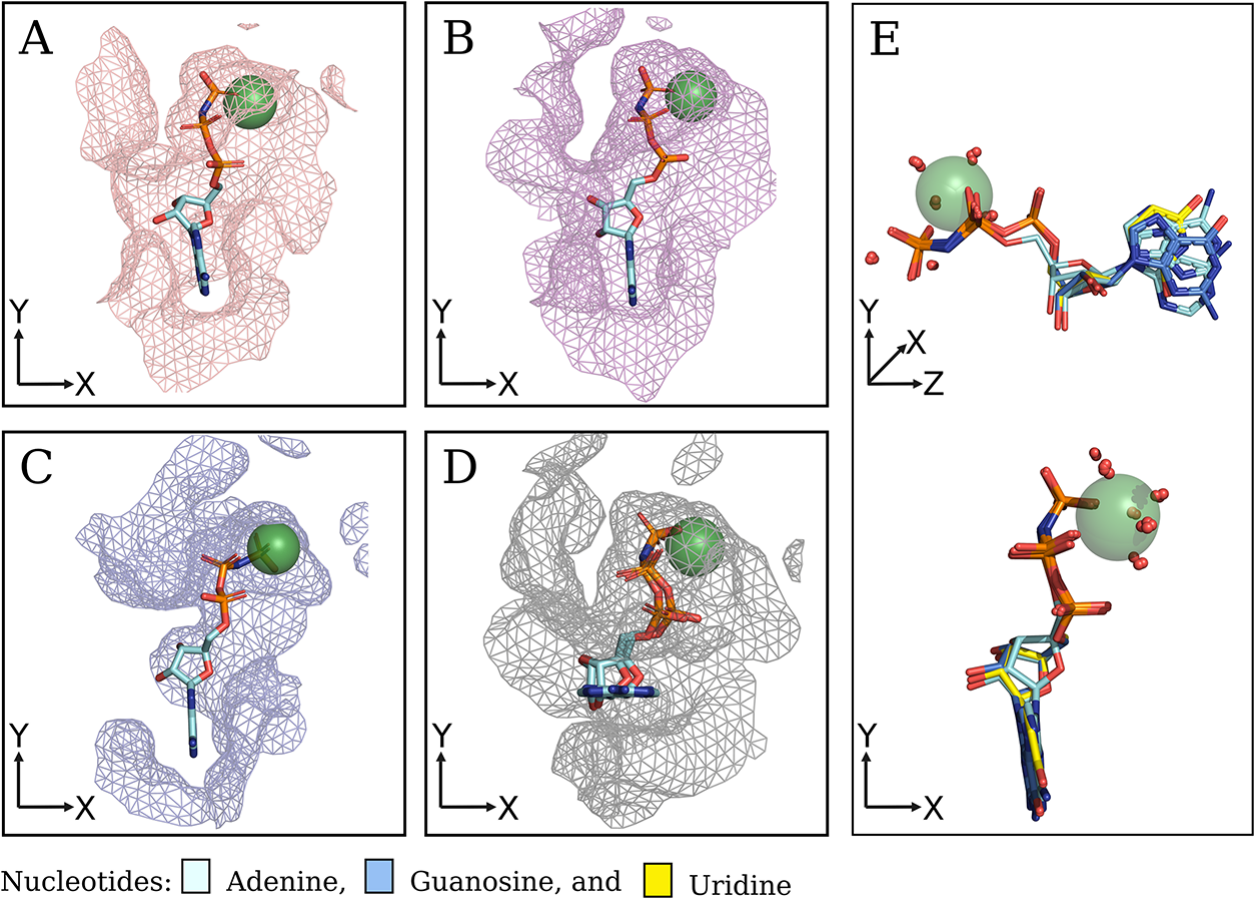
Binding mode of ATP analogs on multiple NTPDases members. The substrate accommodation within the NTPDases active site targets resolved structures with the ATP analog. The active site is portrayed as a mesh surface, the cofactor as a green sphere, and the substrates as sticks, with carbon atoms colored per nucleobase type. The figure illustrates the productive binding mode of ATP analogs on (A) RnNTPDase2 (PDB code: 3CJA), (B) LpNTPDase1 (PDB code: 4BRA), (C) TgNTPDase3 (PDB code: 4A5A), and (D) TgNTPDase1 (PDB code: 4KH4). Additionally, (E) presents a structural alignment of all substrate analogs experimentally resolved in complex with the RnNTPDase2. The small red spheres represent water molecules of the cofactor’s first hydration shell and the catalytic waters.

**Table 1:** Substrates Structural Similarity

enzyme/PDB code	corresponding nucleotide	RMSD (Å)
triphosphates (reference: 3CJA)		
RnNTPDase2/4BQZ	GTP	0.28
RnNTPDase2/4BR2	UTP	0.1
LpNTPDase1/4BRA	ATP	1.13
LpNTPDase1/4BRD	ATP	1.07
LpNTPDase1/4BRG	GTP	0.6
LpNTPDase1/4BRK	UTP	1.04
TgNTPDase3/4A5A	ATP	1.22
diphosphate (reference: 4BR0)		
LpNTPDase1/4BRC	ADP	0.32
LpNTPDase1/4BRL	GDP	0.25
LpNTPDase1/4BRE	ADP	0.14

Assuming ATP and ADP analogs cocrystallized with RnNTPDase2 (PDB code: 3CJA and 4BR0, respectively) as benchmark structures for nucleoside tri- and diphosphates, respectively, we evaluate the similarity among cocrystallized structures by using the PyMOL software, measuring the RMSD of NTPs and NDPs compared to the reference structures. The resultant RMSD values are summarized in Table 1.

Among the triphosphate substrates adopting the linear-like conformation, the highest RMSD value is observed when comparing the reference (PDB code: 3CJA) to the TgNTPDase3 ATP analogue (PDB code: 4A5A), with a deviation of 1.22 Å (Figure 2A). In contrast, an RMSD of 0.32 Å was obtained when aligning the diphosphate reference structure with the ADP analogue bound to LpNTPDase1 (PDB code: 4BRC), representing the highest value among nucleoside diphosphates (Figure 2B). Figure 1E shows the alignment of ATP, ADP, GTP, and UTP analogs cocrystallized with RnNTPDase2, showcasing a nearly identical conformation across different substrate types including the conserved bidentate interaction with the cofactor and the presence of relevant water for the catalytic complex. Due to its frequent occurrence and similarity in multiple crystal structures regardless of the NTPDase isoform or organism or origin, we defined this linear-like substrate conformation of NTPs and NDPs as the canonical substrate structure upon binding to an NTPDase (Figure 2A and B).

**Figure 2:**
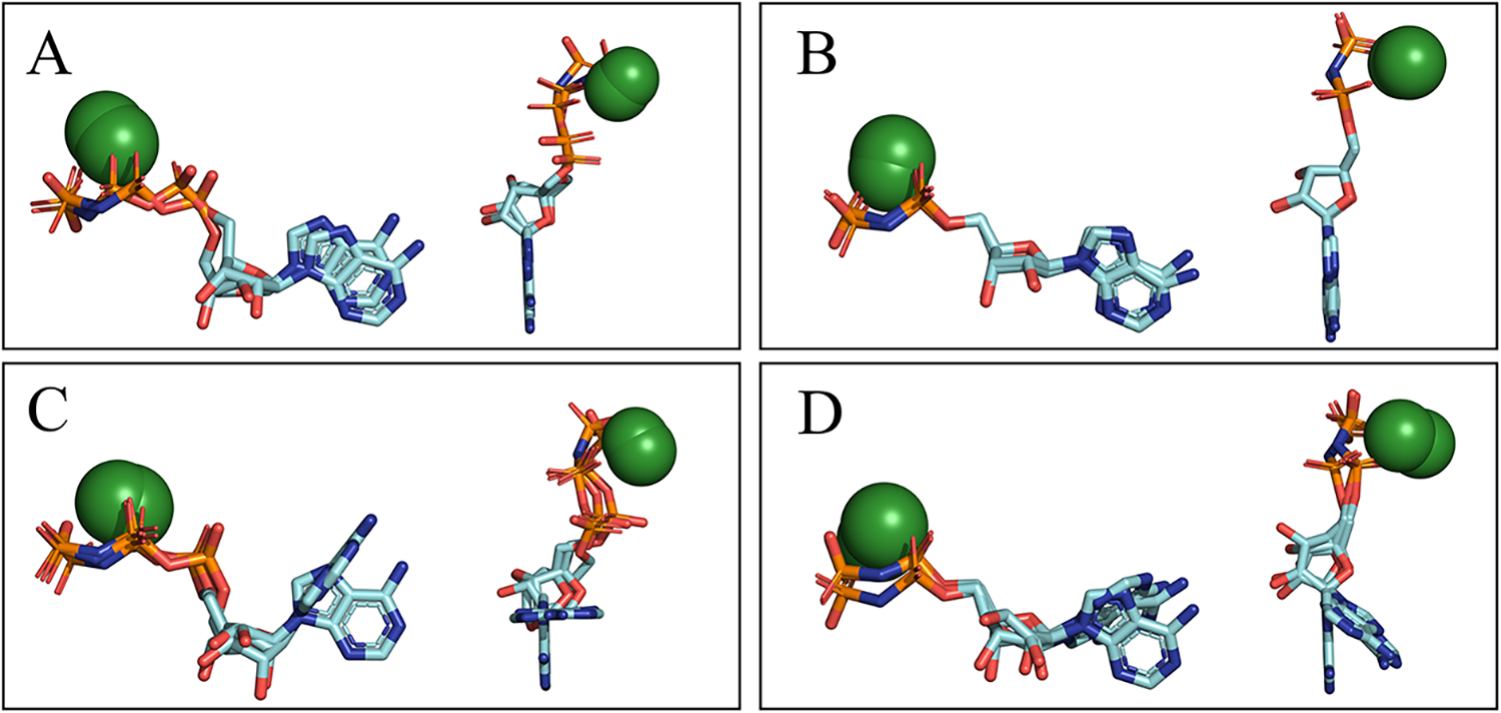
Structural alignment of substrate analogs in different NTPDases highlights the linear-like substrate conformation of NTPs and NDPs as the canonical substrate structure upon binding to an NTPDase. The figures display the structural alignment of the nucleoside triphosphates (A) and diphosphates (B) in the canonical conformation of NTPDases, with the highest RMSD values compared to their respective references: (A) Triphosphate reference (PDB code: 3CJA) to the TgNTPDase3 ATP analogue (PDB code: 4A5A); (B) diphosphate reference structure (PDB code: 4BR0) with the ADP analogue bound to LpNTPDase1 (PDB code: 4BRC). Figures (C, D) show the structural alignment of the alternative binding mode of ATP and ADP analogs, respectively, observed in TgNTPDase1. To enhance visualization of the similarities and differences, Figure 2 A–D present two perspectives of the same alignment per section: one lateral (on the left) and one from above (on the right).

While most NTPDases accommodate the canonical substrate structure, TgNTPDase1 presents an alternative binding mode attributed to a distinct arrangement of residues within its binding site,^12^ as shown in Figure 1D. Notably, TgNTPDase1 is the sole resolved NTPDase structure that physically obstructs the space necessary to establish the canonical linear-like substrate structure. The nucleobase of the ATP analogue observed upon binding to TgNTPDase1 (PDB code: 4A5A) aligns with the *X*-axis. It forms an approximate 90° angle with the nucleobase of the reference NTP structure (Figure 2C). A similar pattern, albeit with a less pronounced angle, is noted when comparing the ADP analogue cocrystallized with the same enzyme and its reference structure (Figure 2D). Nevertheless, despite this divergent orientation of the nucleobase in the TgNTPDase1 complexes, the phosphate and ribose moieties of ATP and ADP analogs still can be aligned (all atom alignment) with RMSD values of 1.64 and 0.64 Å, respectively, when compared to their respective reference structures.

For the LpNTPDase1-UTP analogue complex, two PDB structures report different binding modes. One presents the canonical substrate structure (PDB code: 4BRK), and the other an alternative-like binding mode (PDB code: 4BRI) (Figure S3). As presented in Figure 1B, the binding site of LpNTPDase1 does not physically obstruct the substrate from adopting a linear-like conformation. Additionally, the positioning of the ribose in the alternative-like structure differs from the alternative binding mode characterized in TgNTPDase1. Consequently, the significance of this ambiguous UTP binding conformation in LpNTPDase1 remains to be determined.

Despite variations in substrate conformation or binding mode, certain general features persist in all crystal structures of the productive NTPDase-substrate complexes. These include (1) the bidentate interaction of the cofactor with the nucleotides and (2) a symmetric interaction between the aspartic acid of the DXG motifs on ACRs 1 and ACR4 and the cofactor ion. Excluding the abnormal TgNTPDase1 measurements, when calculated from the DXG aspartic acid’s Cγ, the average distances are 4.80 and 4.84 Å, respectively. Individual measurements for each experimental structure are presented in Table S2. (3) The presence of four water molecules in the cofactor’s first hydration shell, a catalytic water, and an additional water molecule close to the last phosphate group of NTPs and NDPs is also conserved in all crystal structures where the water molecules’ positions were determined (which excludes the TgNTPDase1 and −3 complexes). (4) Regarding the ribose configuration, substrates cocrystallized with RnNTPDase2 consistently exhibit a C2′ endo configuration, while the C3′ endo conformation predominates in other structures.

The productive NTPDase-nucleotide complex is defined by conserved interactions, including hydrogen bonds between the enzyme and nucleoside phosphate, precise positioning of the cofactor, and the presence of six conserved water molecules within the active site.^10^ This binding mode, as characterized in the crystal structure of RnNTPDase2, likely represents the precatalytic conformation of these complexes. Consequently, it serves as a valuable reference for assessing unknown productive NTPDase-substrate complexes.^9^

The structural analysis presented here underscores the high degree of conservation in the substrate structure upon binding across various NTPDases. Thus, it is possible to assume that HsNTPDase-substrate complexes also exhibit these common characteristics. The current experimental structures suggest that without physical obstructions like those seen in TgNTPDase1, different nucleotides will likely adopt the canonical conformation when productively bound to an NTPDase. In light of this context, computational studies aiming to characterize or investigate productive NTPDase-substrate complexes should be based on initial structures that reflect such catalytically relevant configurations.

The exploration of computational methods for studying the structure and functions of NTPDases remains relatively unexplored. To the best of our knowledge, only two previous works have ventured into applying Molecular Docking and Molecular Dynamics (MD) simulations to investigate human NTPDases bound to substrates.^26,27^ In both works, docking calculations were used to model the NTPDase-substrate complex. However, as illustrated in Figure S1, using RMSD values obtained from molecular docking calculations (usually, RMSD lower than 2 Å) may not capture the structurally conserved features observed in experimental structures discussed above, highlighting the limitation of using RMSD as the only quality metric. Such inaccuracies are likely to bias following computational studies such as MD simulations, which are highly dependent on the initial coordinates of the simulated system.^28^

### 2.2 Application of the Proposed Substrate Transferring Protocol to Human NTPDase1–8

To overcome the limitations of our docking attempts, we employed our modeling protocol, detailed in the Methods section. Briefly, the HsNTPDase1–8 were modeled using the AlphaFold2 through the ColabFold engine,^29^ with RnNTPDase2 (PDB code: 3CJA) serving as a custom reference structure.^9^ The rationale behind utilizing a reference structure was to bias the modeling process toward a specific state, that is, the closed NTPDase conformation associated with a productive binding mode.^10^ To ensure that a closed state was modeled, we structurally aligned the enzymes with the template structure (PDB code: 3CJA), evaluating the overall RMSD and the position of ACR1 and 4, which can be used visually as key regions to differentiate between closed and open NTPDase states (Figure S2). As depicted in Figure S4, the modeled structures nicely match the template, thus, confirming a closed state for all of them. These models were then used to build complexes and describe the complexes. Further characterization of the conformational changes associated with each HsNTPDase will be discussed elsewhere (manuscript in preparation).

Following the previously characterized phylogenetic relationship, the enzymes are presented and discussed in pairs.^24^ The following figures, presented from a comparable perspective, highlight the interactions of each enzyme with the ribose and nucleobase of the studied substrates. As previously characterized, the phosphate moiety of all nucleotides engages in highly conserved hydrogen bonds with the catalytic site formed by the ACR1–5.^10^ The Supporting Information (Figures S5–S12) shows these conserved hydrogen bonds to enhance clarity and focus on the more distinguishing features of each complex. Table S3 presents the RMSD values of each substrate compared to the NTP and NDP reference structures and the distance values between the aspartate on each DXG motif and the cofactor.

#### 2.2.1 HsNTPDase1 and HsNTPDase8

While HsNTPDase 1 hydrolyzes NTPs and NDPs with very similar rates, HsNTPDase 8 is more active toward NTPs than NDPs, a feature shared with several cell surface-located NTPDases in different organisms, although each isoform displays unique characteristics. While HsNTPDase1 has a higher activity toward adenine nucleotides and a similar hydrolytic capability when processing NTPs and NDPs, HsNTPDase8 is more active toward NTPs over NDPs regardless of the nucleobase type.^24,30–33^

Our results show that in HsNTPDase1, the stabilization of purine nucleotides in the bound state is mediated by a π-π-π stacking interaction between Phe365, Tyr408, and the substrate’s nitrogenous base (Figure 3A, B, D, and E). Conversely, pyrimidine nucleotides such as UTP and UDP can engage in only one π-π stacking interaction with Phe365 (Figure 3C and F). In comparison, HsNTPDase8 interacts with all substrate bases through a single π-π stacking interaction via the Tyr357 residue. While Trp398 replaces Tyr408 in HsNTPDase1, our results did not indicate a clear interaction between the residue and any substrate (Figure 3G–L). Once in solution, however, conformational changes may place Trp398 in an optimal position to form another π-π interaction with substrates.

**Figure 3:**
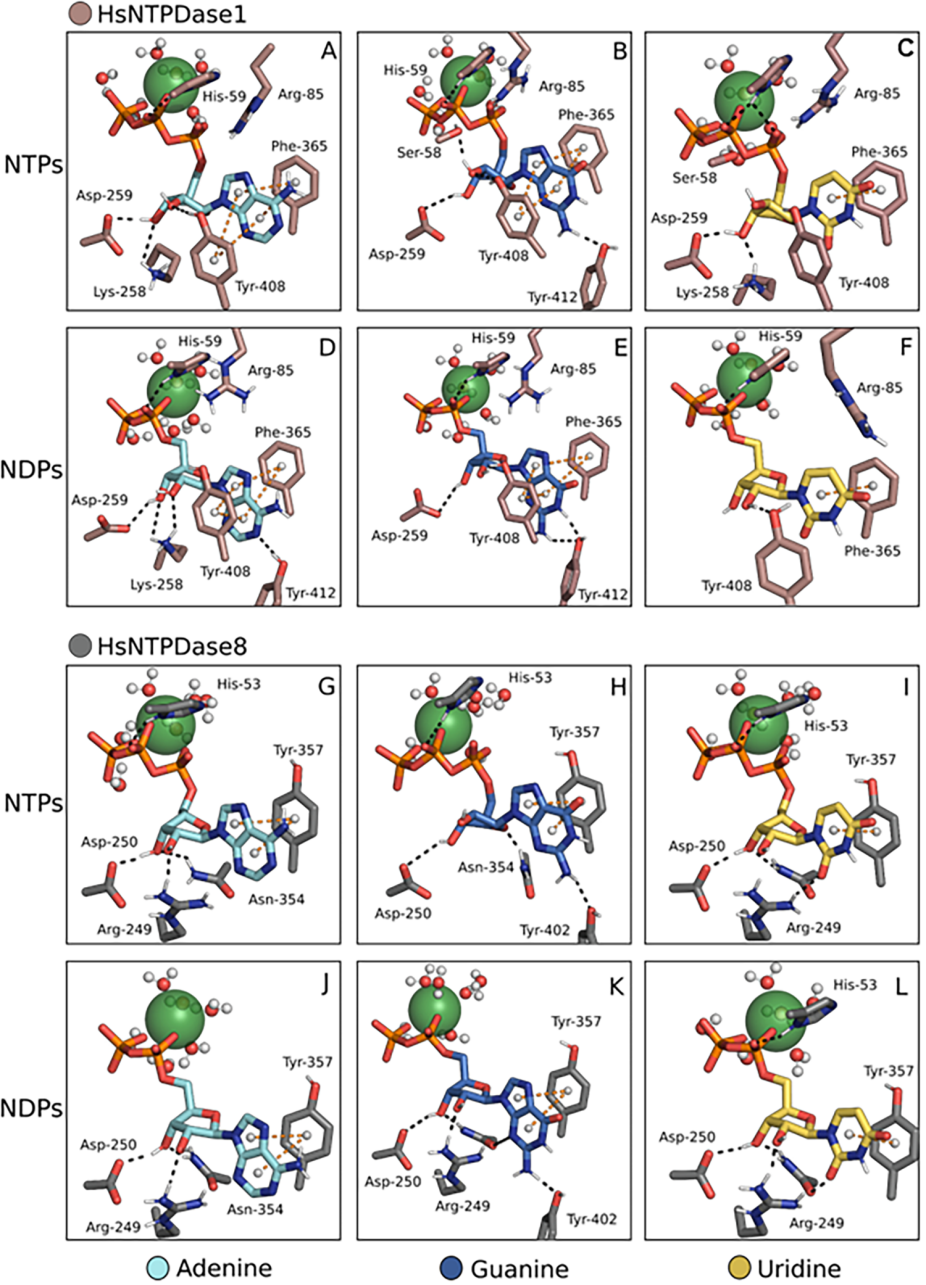
HsNTPDase1 and −8 in complex with multiple substrates. NTPDase-substrate complexes were assembled by the proposed method. HsNTPDase1 is in complex with ATP, GTP, UTP, ADP, GDP, UDP (A–F), and HsNTPDase8 with the same substrates (G–L). Ca^2+^ represented as a green sphere, was the cofactor of choice.

Multiple residues in both enzymes form hydrogen bonds with the ribose substrates. Our model shows that Asp259 and Lys258 interact with both ATP and ADP in HsNTPDase1. Additionally, ATP forms a hydrogen bond with Tyr408, and ADP forms one with Tyr412 through the N1 atom of the adenine base (Figure 3A and D). Both guanine nucleotides form a hydrogen bond with Tyr412. Upon binding to an NTPDase, nucleoside diphosphates such as ADP adopt an elongated structure and the hydrogen bond with the nitrogenous base and others shared with ATP may help stabilize this conformation. GTP and UTP form a hydrogen bond with Ser58 of the ACR1 residue, which is predicted to interact with the phosphate moiety (Figure 3C and D). The competition between the ribose and the phosphates for the same interaction partner could cause instability in these complexes. In contrast, in HsNTPDase8, the enzyme-ribose interactions remain highly conserved, with Arg249, Asp250, and Asn354 forming similar hydrogen bonds with all nucleotides (Figure 3 G–L).

Although not predicted to interact with any nucleotide modeled in this work, the Arg85 residue in HsNTPDase1 is consistently located close to the substrate in all complexes, which is a unique feature of this enzyme (Figure 3A–F). Arg85 is a positively charged amino acid that could interact with NTPs and NDPs’ phosphate moiety during enzyme dynamics and can be an important residue for HsNTPDase1′s similar hydrolysis rates for tri- and diphosphate substrates. Interestingly, in the intracellular HsNTPDase isoforms an arginine immediately after ACR1 occupies a similar spatial region (even though it is not at the equivalent sequence position) and has been shown to confer enhanced NDP specificity.^34^ By analogy, we propose that Arg85 in HsNTPDase1 plays a parallel role: it stabilizes diphosphate binding in the active site and thus helps balance the enzyme’s activity toward both NTPs and NDPs, a feature that distinguishes HsNTPDase1 from the other members of the family.

#### 2.2.2 HsNTPDase2 and HsNTPDase3

The other two cell surface-located NTPDases found in humans are NTPDase2 and NTPDase3. While closely related to the other members of this group, isoforms 2 and 3 exhibit unique characteristics and are identified in their respective branches on the phylogenetic tree.^24^ Regarding their activity, HsNTPDase3 has a standard preference for NTPs over NDPs. Compared to other cell surface NTPDases, HsNTPDase2 stands out as the isoform with the highest preference for nucleoside triphosphates.^24,30^

In HsNTPDase2, Arg394 is functionally similar to HsNTPDase1 Tyr408 in stabilizing substrate bases. This is accomplished through cation-π and π-π interactions mediated by the Tyr350 residue (Figure 4A–F). Conversely, HsNTPDase3 employs Tyr417 and Tyr375 residues to establish the standard π-π stacking interaction with the substrate bases (Figure 4G–L). In the context of hydrogen bonds involving the ribose of the nucleotides, HsNTPDase2 forms four hydrogen bonds with ATP via residues Arg245 and Asp246 (Figure 4A). These identical residues form fewer hydrogen bonds with other substrates (Figure 4B–F). In contrast, HsNTPDase3 engages in fewer interactions with the ribose moiety, with each adenine nucleotide forming two bonds (Figure 4G and J).

**Figure 4:**
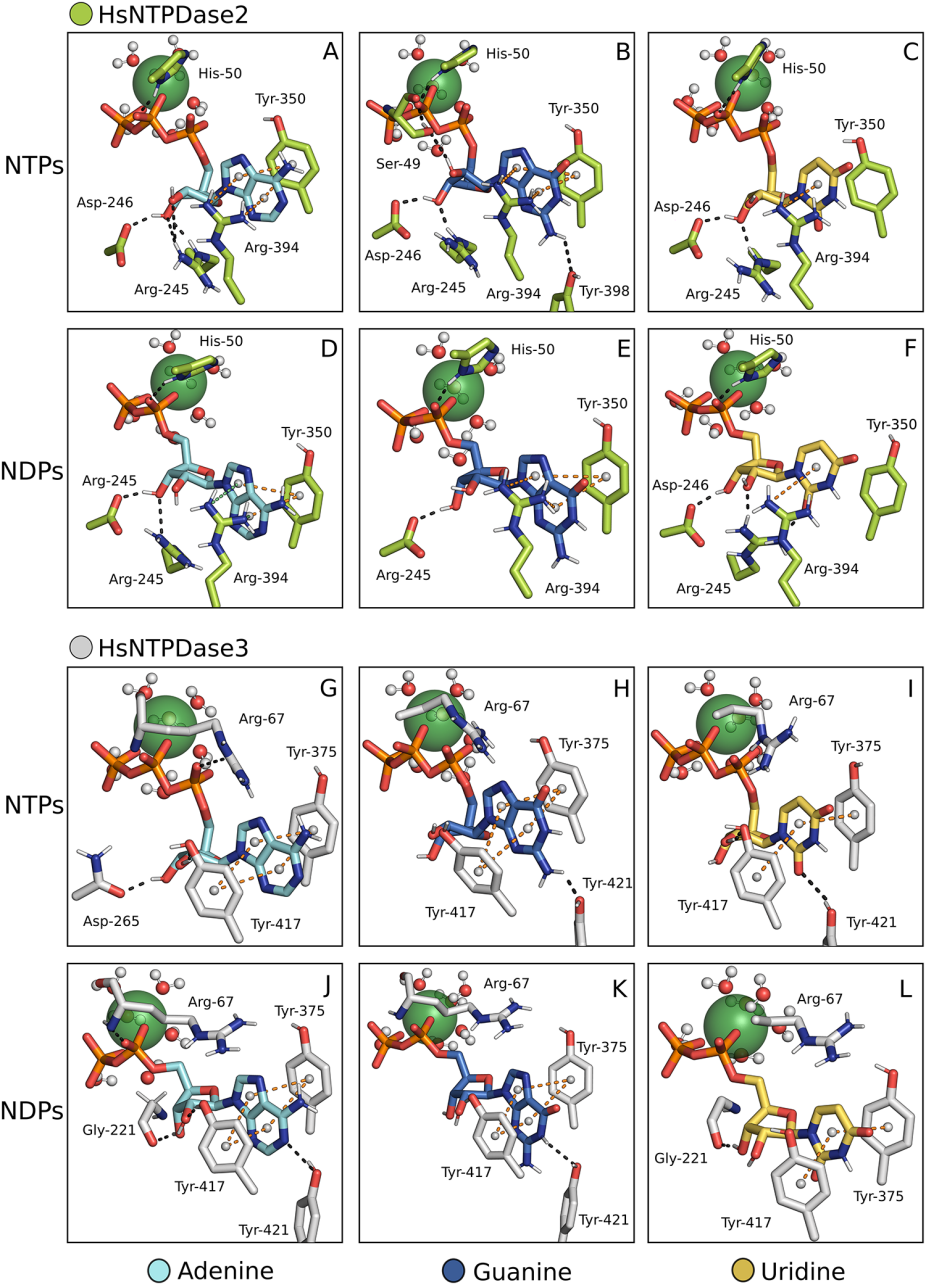
HsNTPDase2 and −3 in complex with multiple substrates. NTPDase-substrate complexes assembled by the proposed method. HsNTPDase2 is in complex with ATP, GTP, UTP, ADP, GDP, UDP (A–F), and HsNTPDase3 with the same substrates (G–L). Ca^2+^, represented as a green sphere, was the cofactor of choice.

Notably, an arginine on ACR1 (Arg67) distinguishes HsNTPDase3 from other cell surface HsNTPDases that possess a histidine residue at the sixth position of ACR1. The histidine residue in other enzymes forms a hydrogen bond between its Nδ and an oxygen atom of the Pβ or Pα in NTPs or NDPs complexes, respectively (Figure 3A–L). In HsNTPDase3, the Arg67 backbone nitrogen forms a hydrogen bond with the substrates, similar to the interaction mediated by the histidine residue in other HsNTPDases (Figure 4G–L).

#### 2.2.3 HsNTPDase4 and HsNTPDase7

Notably, the members of the E-NTPDase family exhibit a highly conserved structure, but sequence conservation is typically low, even among isoforms of the same species. For instance, when human NTPDase isoforms are compared, HsNTPDase4 and HsNTPDase7 demonstrate the highest sequence similarity, sharing up to 67% of their ectodomain sequence. Notably, both enzymes favor uridine substrates, but HsNTPDase4 is more active toward UDP,^35^ whereas HsNTPDase7 is more active toward UTP.^36^ Furthermore, both HsNTPDase4 and 7 stabilize the nitrogenous base of their nucleotides by employing a single π-π interaction with the nucleobase, which is mediated by Tyr436 (Figure 5A–F) and Tyr374 (Figure 5G–L) residues, respectively.

**Figure 5:**
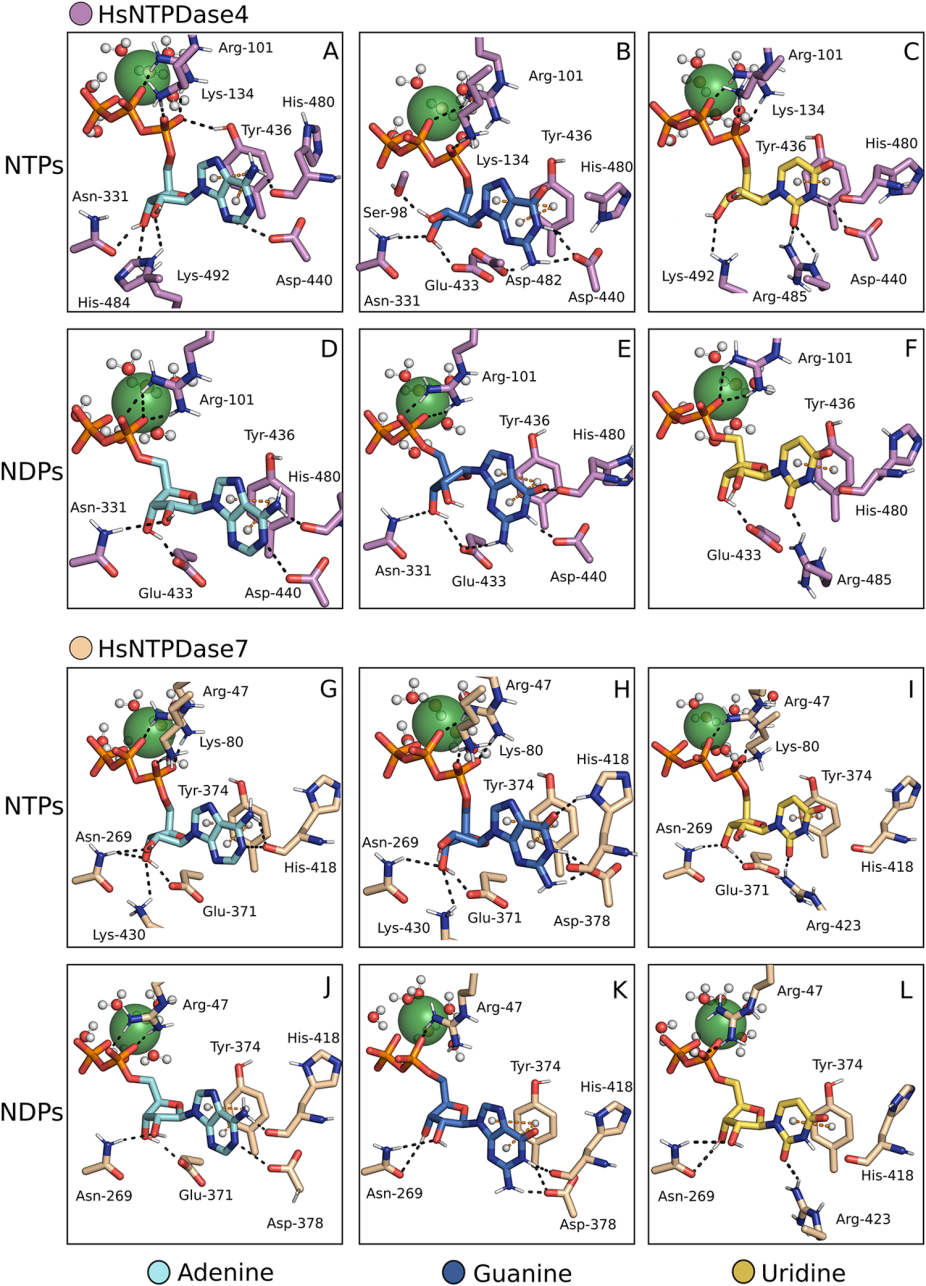
HsNTPDase4 and −7 in complex with multiple substrates. NTPDase-substrate complexes assembled by the proposed method. HsNTPDase4 is in complex with ATP, GTP, UTP, ADP, GDP, UDP (A–F), and HsNTPDase7 with the same substrates (G–L). Ca^2+^, represented as a green sphere, was the cofactor of choice.

Compared with other HsNTPDase residues, these tyrosine residues are found closer to the catalytic site, facilitating the formation of π-π interactions with pyrimidine substrates. Unlike other isoforms, HsNTPDase4 and 7 promote multiple hydrogen bonds with all substrates’ nucleobases (Figure 5A–L). The interactions between these residues may play a crucial role in their specific characteristics.

The nucleotide-binding sites of these two enzymes contain conserved residues that interact with the ribose part of the nucleotides. In HsNTPDase4, residues Asn331, Glu433, and Lys492 mediate this interaction (Figure 5A–F). On HsNTPDase7, the corresponding residues Asn269, Glu370, and Lys430 are responsible for forming the mentioned interaction (Figure 5G–L). His484 also plays a role in substrate interaction in the HsNTPDase4-ATP complex.

All intracellular HsNTPDases share a common characteristic: the presence of an arginine residue, immediately after ACR1. This residue interacts with the phosphate moiety of all nucleotides (Figures 5 and 6 A–L). Based on the information gathered from enzymes related to the NTPDase family, an arginine residue at this position might be a crucial aspect of NDPase activity displayed by these enzymes. Supporting this idea, it has been found that adding an arginine in the corresponding spot of HsNTPDase3 enhances its diphosphatase activity.^34^

**Figure 6:**
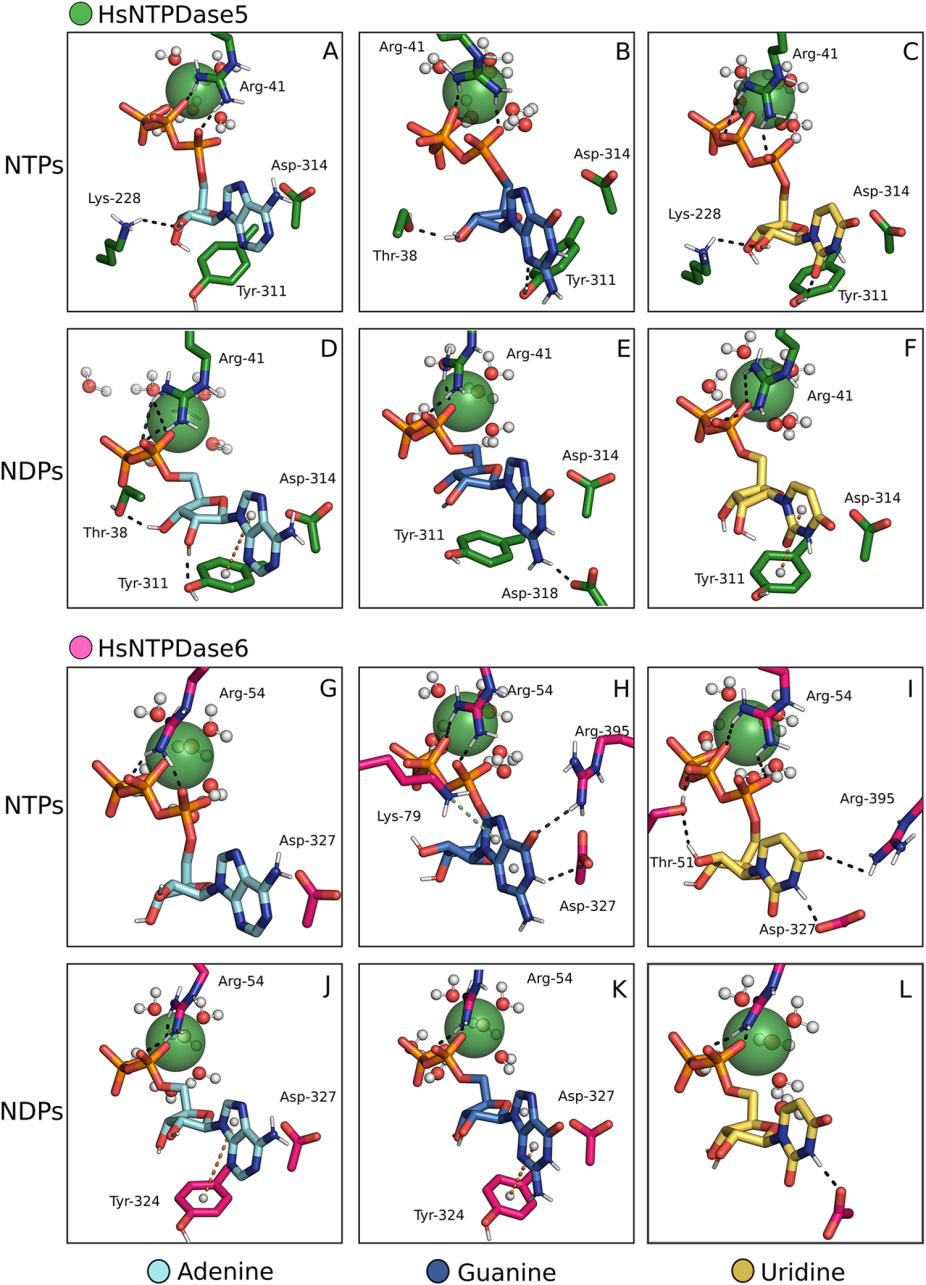
HsNTPDase5 and −6 in complex with multiple substrates. NTPDase-substrate complexes assembled by the proposed method. HsNTPDase5 is in complex with ATP, GTP, UTP, ADP, GDP, UDP (A–F), and HsNTPDase6 with the same substrates (G–L). Ca^2+^, represented as a green sphere, was the cofactor of choice.

The reduced activity of HsNTPDase4 toward adenine nucleotides is another notable feature of the enzyme.^35,37^ It has been suggested that this characteristic may be linked to steric impediments imposed by His480.^16^ The same can be implied for HsNTPDase7 via the corresponding residue His418. In our models, these residues’ positions do not obstruct any substrate. The His480 and His418 form hydrogen bonds with multiple substrates, including ATP and ADP (Figure 5A, B, D, E). However, the dynamical movement of these histidine side chains toward an adenine substrate might elicit a repulsive response between the imidazolium ring of both residues and the adenine amino group. Conversely, guanine and uridine nucleotides might have an opposite response due to the carboxyl groups on both bases. Figure 5H shows a hydrogen bond between the His418 side chain and the ATP carboxyl group.

#### 2.2.4 HsNTPDase5 and HsNTPDase6

The complexes with HsNTPDase5 and −6, despite having a substrate structure resembling crystallographic information, deviate from those formed with other HsNTPDases. Through Tyr311, isoform 5 engages in a T-shaped π-π interaction with the nucleotide bases in ADP and UDP complexes (Figure 6D and F). Asp327 forms a hydrogen bond with uridine nucleotides (Figure 6G–L), while Tyr324 forms T-shaped π-π interactions with ADP and GDP in the HsNTPDase6 complexes (Figure 6G and K). Unlike other isoforms, no hydrogen bonds are formed between the ribosomes of substrates and the enzymes.

The energy minimization and equilibration protocols were applied to each complex to achieve the most energetically favorable complex. However, the standard energy minimization protocol for the other complexes did not accommodate the ATP molecule in the HsNTPDase5 active site. The standard minimization protocol could be applied efficiently only after minimizing the system in a vacuum. Although a direct correlation can not be established, previous studies on HsNTPDase5 have indicated little to no activity toward ATP, suggesting that the formation of a productive ATP-HsNTPDase5 complex is not a favorable process.^38,39^

## 3 Conclusions

The analysis of NTPDase-substrate complexes across various isoforms reveals a highly conserved substrate conformation upon binding to several NTPDases, with most structures exhibiting a canonical anticonfiguration of the nucleoside in a linear-like arrangement of phosphates and nitrogenous base. Variations in substrate structure while maintaining fundamental enzyme–substrate interactions, as the alternative binding mode seen in TgNTPDase1, underscore that the adaptability of the NTPDases to process multiple substrates is likely linked to the stabilization of a conserved nucleotide structure upon binding. This structural conservation suggests that unknown NTPDase-substrate complexes share these features, providing a foundation for computational modeling efforts.

In light of these observations, we propose that transferring cocrystallized substrate-cofactor complexes and important water molecules to well-modeled NTPDase structures offers a reliable method for modeling NTPDase-substrate complexes. Refining these complexes through energy minimization algorithms and restrained MD simulations allows for adaptation of the enzyme and transferred molecules to one another. This method ensures that the conserved features observed in experimental structures are accurately reproduced, enhancing the reliability of computational predictions regarding NTPDase-nucleotide complexes.

We have modeled and described HsNTPDase1–8 with multiple substrates, demonstrating the utility of our approach. Although a static model limits the depth of information accessible, these structures serve as literature-consistent starting points for further characterization studies using MD simulations or QM/MM calculations. Research groups are encouraged to use the three-dimensional (3D) coordinates of all systems studied here, which can be freely obtained at https://github.com/LIMA-UFV/HsNTPDase-Paper. Further investigation utilizing these models can lead to a better understanding of the HsNTPDases specificities, potentially providing the necessary information for its optimization and unleashing the therapeutic potential of these enzymes.

## 4 Methods

### 4.1 Structural Alignment of Cocrystallized Substrate Analog and Analysis

Considering the Adenosine-5″-[(β, γ)-imido]­triphosphate (AMP-PNP) ATP analogue and the Adenosine-5′-[(α, β)-imido]­diphosphate (AMP-NP) ADP analogs cocrystallized with the RnNTPDase2 (Protein Data Bank (PDB) code: 3CJA and 4BR0, respectively) as reference structures for nucleoside triphosphates and diphosphates upon binding to an NTPDase, respectively, we calculated the Root Mean Square Deviation (RMSD) between pairs of structures using the *align* tool within PyMOL (PyMOL Molecular Graphics System, Version 2.5 Schrödinger, LLC). The alignment was carried out using the entire modeled sequence of each NTPDase (Table S1). As summarized in Table S1, structures of the RnNTPDase2,^9,10^ *L. pneumophila* (Lp) NTPDase1,^10^ and *T. gondii* (Tg) NTPDase1 and −3 ^12,13^ retrieved from the PDB were considered in the analysis of substrate binding mode.^40^ Distance calculations between active site residues and substrate atoms were also performed by using PyMOL.

### 4.2 HsNTPDases Molecular Modeling

The reference sequence of each HsNTPDase used in the modeling process was obtained from the UniProt database.^41^ To identify the transmembrane or signal peptide regions in the sequence, we used the DeepTMHMM web server.^42^ Table S1 compiles the UniProt entry code and the modeled sequence regions of each HsNTPDase1–8. The structures of the HsNTPDase1–8 ectodomains were built using the AlphaFold2 engine through the ColabFold AlphaFold2_mmseqs2 Jupyter notebook platform with default settings and using the RnNTPDase2 (PDB code: 3CJA) as a custom template.^18,29^ For each enzyme, five models were generated. Following the automatic quality score classification of the platform, each enzyme’s best model was selected and used in the subsequent steps. To ensure that the models were in a suitable conformation for describing their complex with substrates, we structurally aligned each of the best models with their template PDB entry 3CJA to evaluate the similarity of the structures using PyMOL software. The overall similarity was assessed by measuring the RMSD based on the α-carbon coordinates. As shown in Figure S2, out of the five Apyrase Conserved Regions (ACR1–5), ACR1 and ACR4 relative positions drastically change if the enzyme is open or closed. For this reason, the alignments were also evaluated visually regarding the positioning of the mentioned regions.

### 4.3 Substrate Transferring from Experimental Structures to Modeled HsNTPDases

For each modeled enzyme, we built complexes with the primary nucleotides hydrolyzed by these enzymes: ATP, ADP, GTP, GDP, UTP, and UDP. Using PyMOL, each HsNTPDase was structurally aligned with a reference crystal structure to transfer the substrate from the crystal to the human enzymes. The ATP, ADP, GTP, and UTP substrate structures were extracted from RnNTPDase2 crystals PDB codes 3CJA, 4BR0, 4BQZ, and 4BR2, respectively. To construct GDP and UDP structures, we aligned the aforementioned GTP and UTP substrate analogs with the ADP analogue found in 4BR0 by their nucleobase, saving each mentioned triphosphate structure as a new and aligned PDB file. Based on these properly aligned structures, by directly editing the PDB file information, we build a GDP and a UDP structure in a productive conformation by combining the tridimensional information on the aligned GTP and UTP nucleobase with the sugar and phosphate tail of the ADP analogue found in 4BR0. All of the mentioned crystal structures were crystallized with nonhydrolyzable analogs of each substrate. To reconstruct the substrate molecule, the nitrogen N3β or N3α in the triphosphate and diphosphate analogs was manually substituted into oxygen atoms. In all of the complexes, we used calcium as a cofactor. We also included the four water molecules in the calcium first hydration shell and the two water molecules predicted to have a role in the catalytic process and present multiple crystal structures.^9,10,12^

### 4.4 Energy Minimization and Conformational Equilibration

Before the construction of each MD simulation system, the protonation state of the enzyme–substrate complex was evaluated using the PlayMolecule ProteinPrepare web server.^43^ A simulation system was created for each HsNTPDase-substrate complex using the CHARMM-GUI web server.^44^ The system was solvated in a cubic box with the modified TIP3P water model^45–47^ and neutralized with K^+^ and Cl^–^ to a final concentration of 0.15 M. The CHARMM36m force field was used with the WYF parameters for cation-π interactions.^48,49^ The CHARMM software performed the energy minimization and equilibration protocols.^50^ Energy minimization used the steepest descent and the Adopted Basis Newton–Raphson methods, each for 50 steps.^51,52^ Subsequently, equilibration was performed for 10,000 steps using a 1 fs integration time under an NVT ensemble. Electrostatic interactions were calculated via the Particle Mesh Ewald method^53^ with a real-space cutoff of 12 Å while short-range van der Waals forces were switched smoothly to zero from 10 to 12 Å. To prevent large changes in the complex, energy minimization and equilibration were performed under positional restraints in the backbone and side chain of 1.0 kcal/mol/Å^2^ and 0.1 kcal/mol/Å^2^, respectively. Finally, an energy minimization step was performed (200 steps of steepest descent and 200 steps of ABNR) to resolve geometries that arose from thermal fluctuations during equilibration.

For the HsNTPDase5-ATP complex, an extra vacuum energy minimization was applied before the solvation procedure.

## Supplementary Material



## Notes

Showing the binding mode of RnNTPDase2 predicted by Molecular Docking compared with the crystal structures for ADP and ATP substrates (Figure S1), showing the experimentally characterized closed and open states of NTPDases (Figure S2), showing the alternative-like UTP structure upon binding to LpNTPDase1 (Figure S3), showing modeled HsNTPDases closed state (Figure S4), showing 2D protein–substrate predicted interaction for human NTPDases 1–8 (Figures S5–S12), showing the modeled enzymes (Table S1), showing the list of experimental structures with a productive substrate binding mode (Table S2), and showing the measurements and interaction of the transferred substrate to HsNTPDases (Table S3) (PDF)

## References

[1] Knowles A. F. (2011). The GDA1_CD39
superfamily: NTPDases with diverse functions. Purinergic Signalling.

[2] Burnstock G., Satchell D. G., Smythe A. (1972). A comparison of the
excitatory and
inhibitory effects of non-adrenergic, non-cholinergic nerve stimulation
and exogenously applied ATP on a variety of smooth muscle preparations
from different vertebrate species. Br. J. Pharmacol..

[3] Sévigny, J. Encyclopedia of Signaling Molecules. In Encyclopedia of Cancer, 2nd ed.; Choi, S. , Ed.; 2018; pp 1544–1553.

[4] Gomes-vieira L., Meyer-fernandes R. (2021). E-NTPDases : Possible Roles on Host-Parasite
Interactions and Therapeutic Opportunities. Front. Cell. Infect. Microbiol..

[5] da
Silva W., Ribeiro I. C., Agripino J., de M. (2023). Leishmania infantum NTPDase1 and NTPDase2 play an important role
in infection and nitric oxide production in macrophages. Acta Trop..

[6] Dwyer K. M., Deaglio S., Gao W. (2007). CD39 and control of
cellular immune responses. Purinergic Signalling.

[7] Liu Y., Li Z., Zhao X. (2023). Review immune response of targeting CD39 in
cancer. Biomark Res..

[8] Baghbani E., Noorolyai S., Shanehbandi D. (2021). Regulation of immune
responses through CD39 and CD73 in cancer: Novel checkpoints. Life Sci..

[9] Zebisch M., Sträter N. (2008). Structural insight into signal conversion
and inactivation
by NTPDase2 in purinergic signaling. Proc. Natl.
Acad. Sci. U.S.A..

[10] Zebisch M., Krauss M., Schäfer P. (2013). Crystallographic snapshots
along the reaction pathway of nucleoside triphosphate diphosphohydrolases. Structure.

[11] Zebisch M., Krauss M., Schäfer P., Sträter N. (2012). Crystallographic
evidence for a domain motion in rat nucleoside triphosphate diphosphohydrolase
(NTPDase) 1. J. Mol. Biol..

[12] Krug U., Totzauer R., Zebisch M., Sträter N. (2013). The ATP/ADP
substrate specificity switch between Toxoplasma gondii NTPDase1 and
NTPDase3 is caused by an altered mode of binding of the substrate
base. ChemBioChem.

[13] Krug U., Zebisch M., Krauss M., Sträter N. (2012). Structural
insight into activation mechanism of Toxoplasma gondii nucleoside
triphosphate diphosphohydrolases by disulfide reduction. J. Biol. Chem..

[14] Vivian J. P., Riedmaier P., Ge H. (2010). Crystal Structure of
a Legionella pneumophila Ecto -Triphosphate Diphosphohydrolase, A
Structural and Functional Homolog of the Eukaryotic NTPDases. Structure.

[15] Summers E. L., Cumming M. H., Oulavallickal T. (2017). Structures and kinetics
for plant nucleoside triphosphate diphosphohydrolases (NTPDases) support
a domain motion catalytic mechanism. Protein
Sci..

[16] Gorelik A., Labriola J. M., Illes K., Nagar B. (2020). Crystal structure of
the nucleotide-metabolizing enzyme NTPDase4. Protein Sci..

[17] Wu L., Qin L., Nie Y. (2022). Computer-aided understanding and engineering
of enzymatic selectivity. Biotechnol Adv..

[18] Jumper J., Evans R., Pritzel A. (2021). Highly accurate protein
structure prediction with AlphaFold. Nature.

[19] Terwilliger T. C., Liebschner D., Croll T. I. (2024). AlphaFold predictions
are valuable hypotheses and accelerate but do not replace experimental
structure determination. Nat. Methods.

[20] Yang F., Hicks-Berger C. A., Smith T. M., Kirley T. L. (2001). Site-directed mutagenesis
of human nucleoside triphosphate diphosphohydrolase 3: The importance
of residues in the apyrase conserved regions. Biochemistry.

[21] Rao L., Jia N. X., Hu J. (2022). ATPdock: A template-based
method for ATP-specific protein-ligand docking. Bioinformatics.

[22] Jakhar R., Dangi M., Khichi A., Chhillar A. K. (2020). Relevance of molecular
docking studies in drug designing. Curr. Bioinf..

[23] Saikia S., Bordoloi M. (2019). Molecular Docking:
Challenges, Advances and its Use
in Drug Discovery Perspective. Curr. Drug Targets.

[24] Robson S. C., Sévigny J., Zimmermann H. (2006). The E-NTPDase family of ectonucleotidases:
Structure function relationships and pathophysiological significance. Purinergic Signalling.

[25] Laliberte J. F., Beaudoin A. R. (1983). Sequential hydrolysis of the gamma-
and beta-phosphate
groups of ATP by the ATP diphosphohydrolase from pig pancreas. Biochim. Biophys. Acta.

[26] Vadlamani V. M. K., Gunasinghe K. K. J., Chee X. W. (2023). Human
soluble CD39 displays substrate inhibition in a substrate-specific
manner. Sci. Rep..

[27] Iqbal J., Shah S. J. A. (2018). Molecular dynamic
simulations reveal structural insights
into substrate and inhibitor binding modes and functionality of Ecto-Nucleoside
Triphosphate Diphosphohydrolases. Sci. Rep..

[28] Hollingsworth S. A., Dror R. O. (2018). Molecular Dynamics
Simulation for All. Neuron.

[29] Mirdita M., Schütze K., Moriwaki Y. (2022). ColabFold: making protein
folding accessible to all. Nat. Methods.

[30] Kukulski F., Lévesque S. A., Lavoie É. G. (2005). Comparative hydrolysis
of P2 receptor agonists by NTPDases 1, 2, 3 and 8. Purinergic Signalling.

[31] Christoforidis S., Papamarcaki T., Galaris D. (1995). Purification and Properties
of Human Placental ATP Diphosphohydrolase. Eur.
J. Biochem..

[32] Fausther M., Lecka J., Kukulski F. (2007). Cloning, purification,
and identification of the liver canalicular ecto-ATPase as NTPDase8. Am. J. Physiol. Gastrointest. Liver Physiol..

[33] Knowles A. F., Li C. (2006). Molecular cloning and characterization
of expressed human ecto-nucleoside
triphosphate diphosphohydrolase 8 (E-NTPDase 8) and its soluble extracellular
domain. Biochemistry.

[34] Moeckel D., Jeong S. S., Sun X. (2014). Optimizing human apyrase
to treat arterial thrombosis and limit reperfusion injury without
increasing bleeding risk. Sci. Transl. Med..

[35] Wang T. F., Guidotti G. (1998). Golgi localization
and functional expression of human
uridine diphosphatase. J. Biol. Chem..

[36] Shi J. D., Kukar T., Wang C. Y. (2001). Molecular Cloning and
Characterization of a Novel Mammalian Endo-apyrase (LALP1). J. Biol. Chem..

[37] Biederbick A., Kosan C., Kunz J., Elsässer H. P. (2000). First apyrase
splice variants have different enzymatic properties. J. Biol. Chem..

[38] Mulero J. J., Yeung G., Nelken S. T., Ford J. E. (1999). CD39-L4 is a secreted
human apyrase, specific for the hydrolysis of nucleoside diphosphates. J. Biol. Chem..

[39] Murphy-Piedmonte D. M., Crawford P. A., Kirley T. L. (2005). Bacterial expression,
folding, purification
and characterization of soluble NTPDase5 (CD39L4) ecto-nucleotidase. Biochim. Biophys. Acta, Proteins Proteomics.

[40] Berman H. M., Westbrook J., Feng Z. (2000). The Protein Data Bank. Nucleic
Acids Res..

[41] Apweiler R., Bairoch A., Wu C. H. (2004). UniProt: The universal
protein knowledgebase. Nucleic Acids Res..

[42] Hallgren, J. ; Tsirigos, K. D. ; Damgaard Pedersen, M. , (2022) DeepTMHMM predicts alpha and beta transmembrane proteins using deep neural networks.

[43] Martínez-Rosell G., Giorgino T., De Fabritiis G. (2017). PlayMolecule ProteinPrepare: A Web
Application for Protein Preparation for Molecular Dynamics Simulations. J. Chem. Inf. Model..

[44] Lee J., Cheng X., Swails J. M. (2016). CHARMM-GUI Input Generator
for NAMD, GROMACS, AMBER, OpenMM, and CHARMM/OpenMM Simulations Using
the CHARMM36 Additive Force Field. J. Chem.
Theory Comput..

[45] Neria E., Fischer S., Karplus M. (1996). Simulation of activation
free energies
in molecular systems. J. Chem. Phys..

[46] Jorgensen W. L., Chandrasekhar J., Madura J. D. (1983). Comparison of simple
potential functions for simulating liquid water. J. Chem. Phys..

[47] Durell S. R., Brooks B. R., Ben-Naim A. (1994). Solvent-induced forces between two
hydrophilic groups. J. Phys. Chem. A.

[48] Huang J., Rauscher S., Nawrocki G. (2017). CHARMM36m: An improved
force field for folded and intrinsically disordered proteins. Nat. Methods.

[49] Khan H. M., MacKerell A. D., Reuter N. (2019). Cation-π Interactions between
Methylated Ammonium Groups and Tryptophan in the CHARMM36 Additive
Force Field. J. Chem. Theory Comput..

[50] Brooks, B. R., III ; CLB, A. D. ; Mackerell, J. R. CHARMM: The Biomolecular Simulation Program J. Comput. Chem. 30 1545 1614 10.1002/jcc.21287. PMC2810661 19444816

[51] Haug E. J., Arora J. S., Matsui K. (1976). A steepest-descent
method for optimization
of mechanical systems. J. Optim. Theory Appl..

[52] Ypma, T. J. Historical development of the newton-raphson method SIAM Rev. 37 531 551 10.1137/1037125.

[53] Essmann U., Perera L., Berkowitz M. L. (1995). A smooth particle mesh
Ewald method. J. Chem. Phys..

