# Supplementary material for: Structural Modeling of NTPDase-Substrate Complexes Preserving Catalytic Experimental Features
Source: ACS Omega. 2025 Sep 22;10(38):43891–902. doi: 10.1021/acsomega.5c04628 (PMC12489842; doi:10.1021/acsomega.5c04628)
Supplement: Supplementary file 1 [file ao5c04628_si_001.pdf]

Structural Modeling of NTPDase-Substrate Complexes Preserving Catalytic Experimental Features

João Victor B. de Moraes[1,2], Marcelo D. Polêto[3], Raissa B. de Castro[4], Gustavo C. Bressan[4], Raphael de S. Vasconcellos[4], Jean Sévigny[5,6*] and Juliana R. Fietto[4,7]

[1] General Biology Department, Universidade Federal de Viçosa, Viçosa, Minas Gerais, 36570 900, Brazil.

[2] Laboratório de Biologia Teórica e Computacional (LBTC), Universidade de Brasília, Brasília, Distrito Federal, 70910-900, Brazil.

[3] Department of Biotechnology, Universidade de São Paulo, Lorena, São Paulo, 12602-810, Brazil.

[4] Biochemistry and Molecular Biology Department, Universidade Federal de Viçosa, 36570 900, Viçosa, Minas Gerais, Brazil.

[5] Département de microbiologie-infectiologie et d'immunologie, Centres PROTEO et ARThrite, Université Laval, Faculté de Médecine, Quebec city, QC G1V 0A6, Canada.

[6] Axe Maladies infectieuses et immunitaires, Centre de recherche du CHU de Québec – Université Laval, Quebec city, QC G1V 4G2, Canada.

[7] Instituto de Biotecnologia Aplicada à Agropecuária (Bioagro), Universidade Federal de Viçosa, Viçosa, Minas Gerais, 36570 900, Brazil.

* The last two authors share senior authorship and serve as corresponding authors.

Phone: +55 (31) 3612-2464

E-mail: jufietto@ufv.br

E-mail: Jean.Sevigny@crchudequebec.ulaval.ca

Supplementary material

To illustrate how Molecular Docking can fall to capture the conserved features in the experimental structures of the NTPDase-substrate complex, we used the DockThor web server to dock ADP and ATP on the RnNTPDase2. We compared the results with the crystal structures. Figures S1B and S1D show the discrepancies between predicted and experimental substrate conformations. Despite the clear difference, the docked and experimental NDP and NTP structures can be aligned with an RMSD of 0.543 and 1.33, respectively.

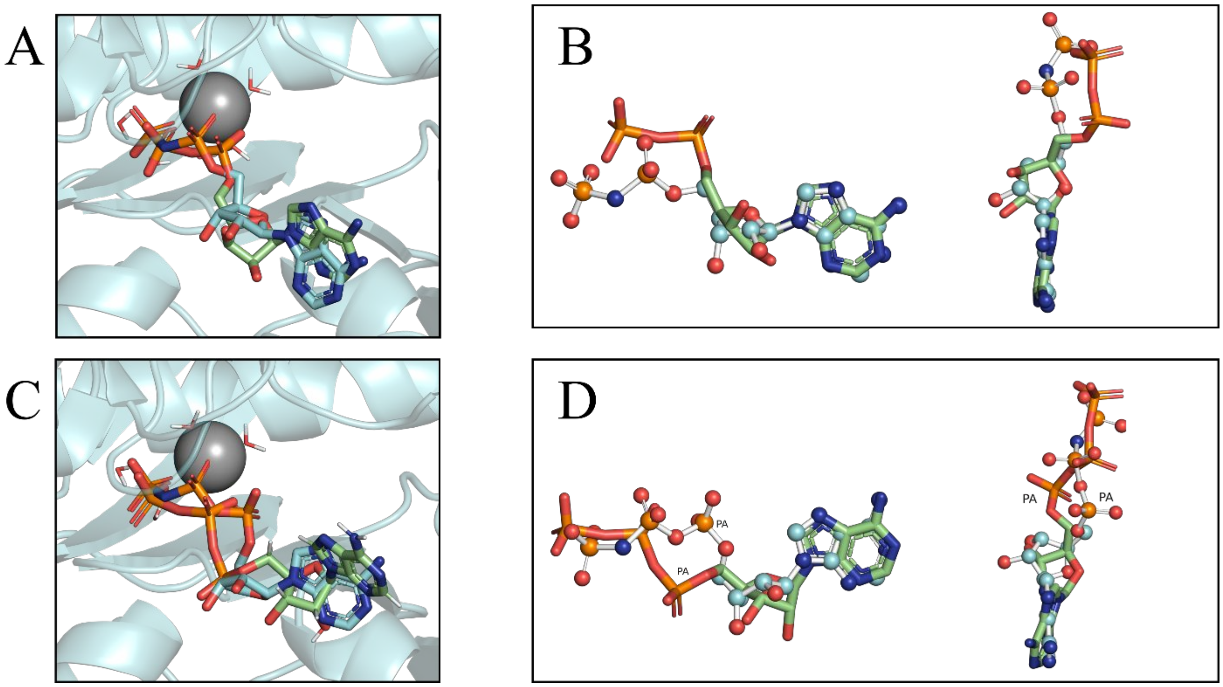

Figure S1 – Binding mode predicted by Molecular Docking compared with the crystal structures for ADP and ATP substrates. The RnNTPDase2 crystal structures 4BR0 and 3CJA

(cartoon) and the co-crystalized ADP and ATP analogs (sticks) are presented in cyan. A and C show the best-predicted pose for ADP and ATP (green sticks in both), respectively, in the RnNTDase2. B and D depict the structural alignment of the best-predicted pose for ADP and ATP (green sticks), respectively, with their corresponding reference nucleotide analog represented as balls and sticks.

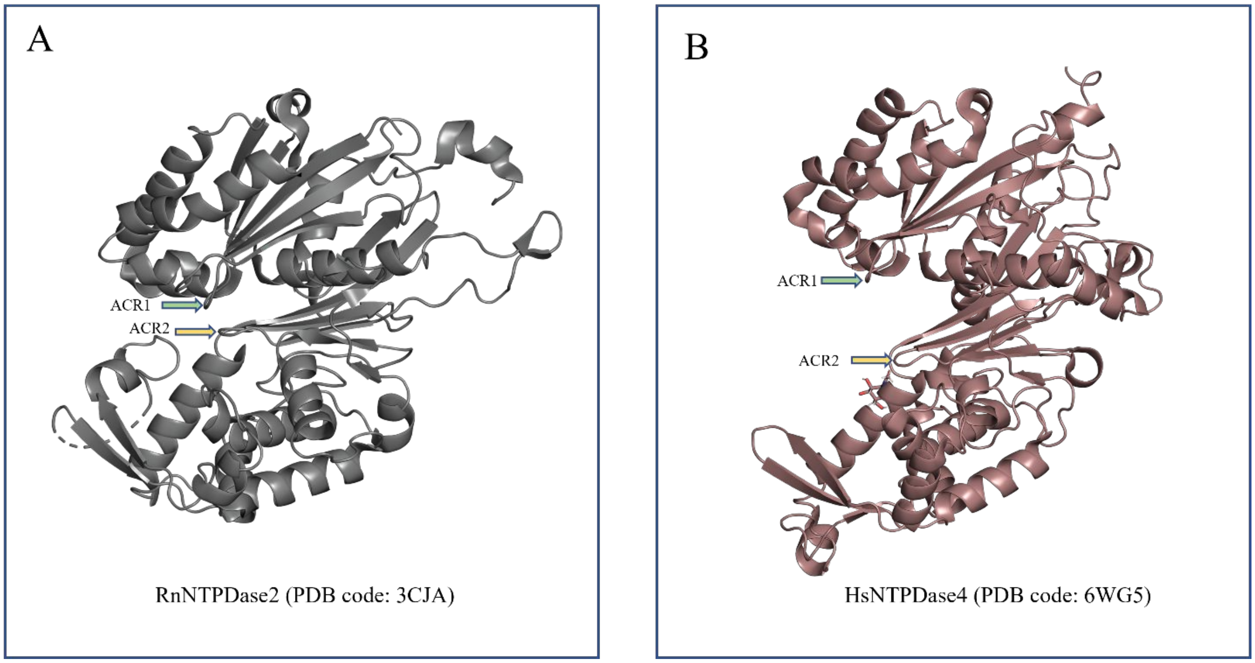

Figure S2 – Experimentally characterized closed and open states. The distance difference between ACR1 and ACR4 in the RnNTPDase2 closed conformation (A) and the HsNTPDase4 open conformation (B). The structures correspond do the PDB code 3CJA and 6WG5, respectively.

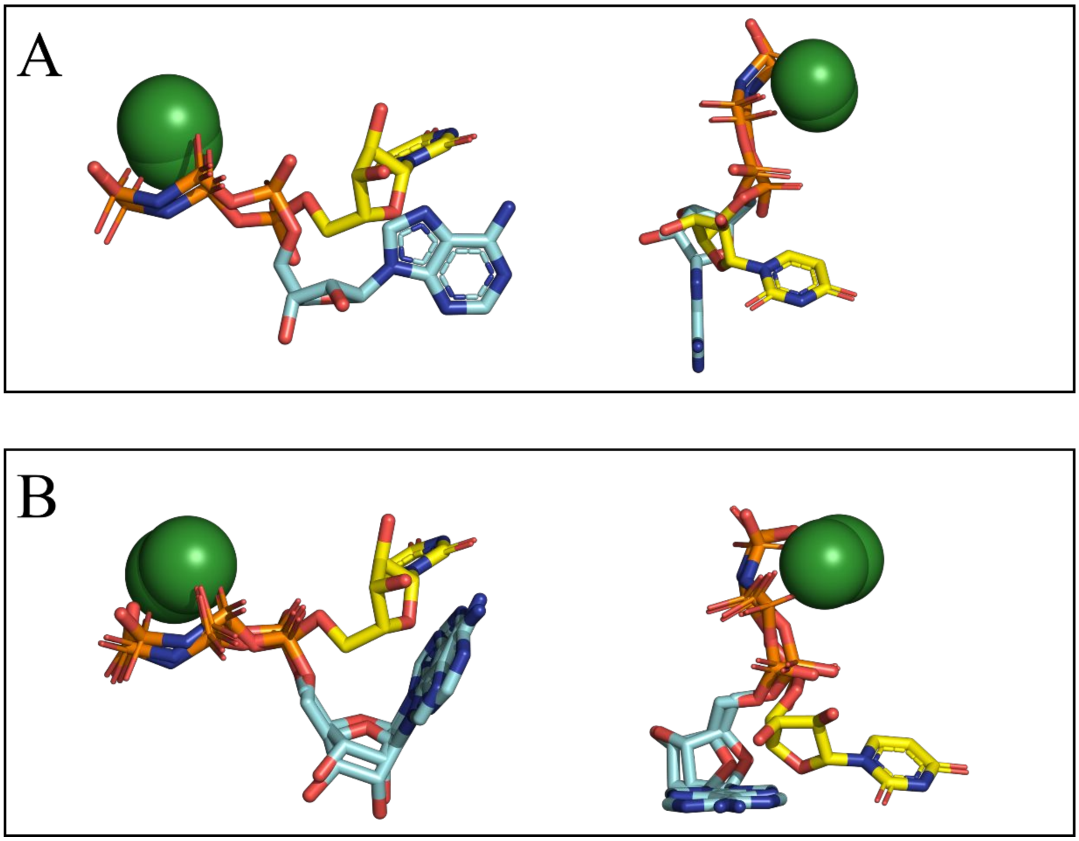

Figure S3 – Alternative-like UTP structure upon binding to LpNTPDase1. The alignment of the UTP alternative-like structure with the reference NTP structure (A) and with the ATP molecule in the alternative binding mode (B) shows the unique features of this unambiguous binding mode. To enhance visualization of the similarities and differences, two perspectives of the same alignment per section are presented: one lateral (on the left) and one from above (on the right).

The alternative-like UTP structure observed with the LpNTPDase1 (PDB code: 4BRI) is a unique conformation that remains to be rationalized. Although the phosphate moiety and cofactor

reproduce the general characteristics observed in other complexes, the ribose is positioned in a unique conformation among all crystal structures.

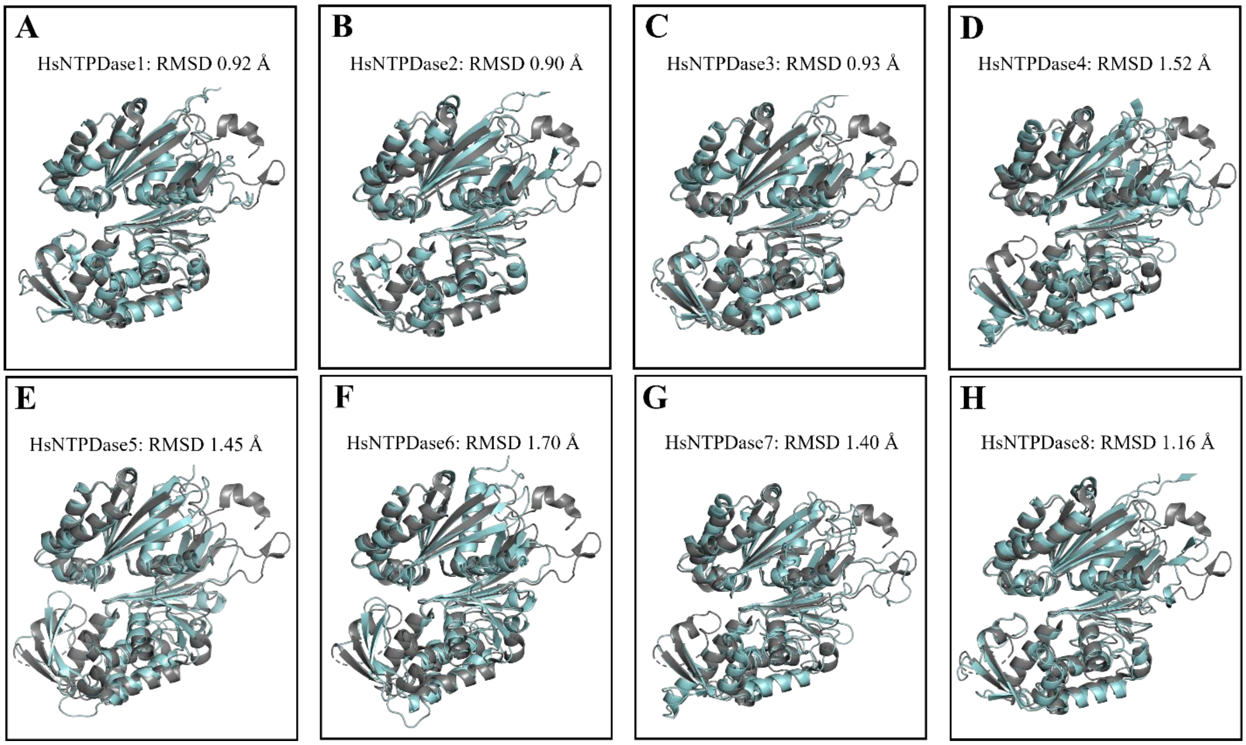

Figure S4 – modeled HsNTPDase closed state. A-H shows the structural alignment of each HsNTPDase best model (cyan) with the RnNTPDase2 (dark gray) experimental structure (PDB code:3CJA). By the similarity of the structures, we can ensure that all of them are in a closed state suitable for describing their complex with substrates.

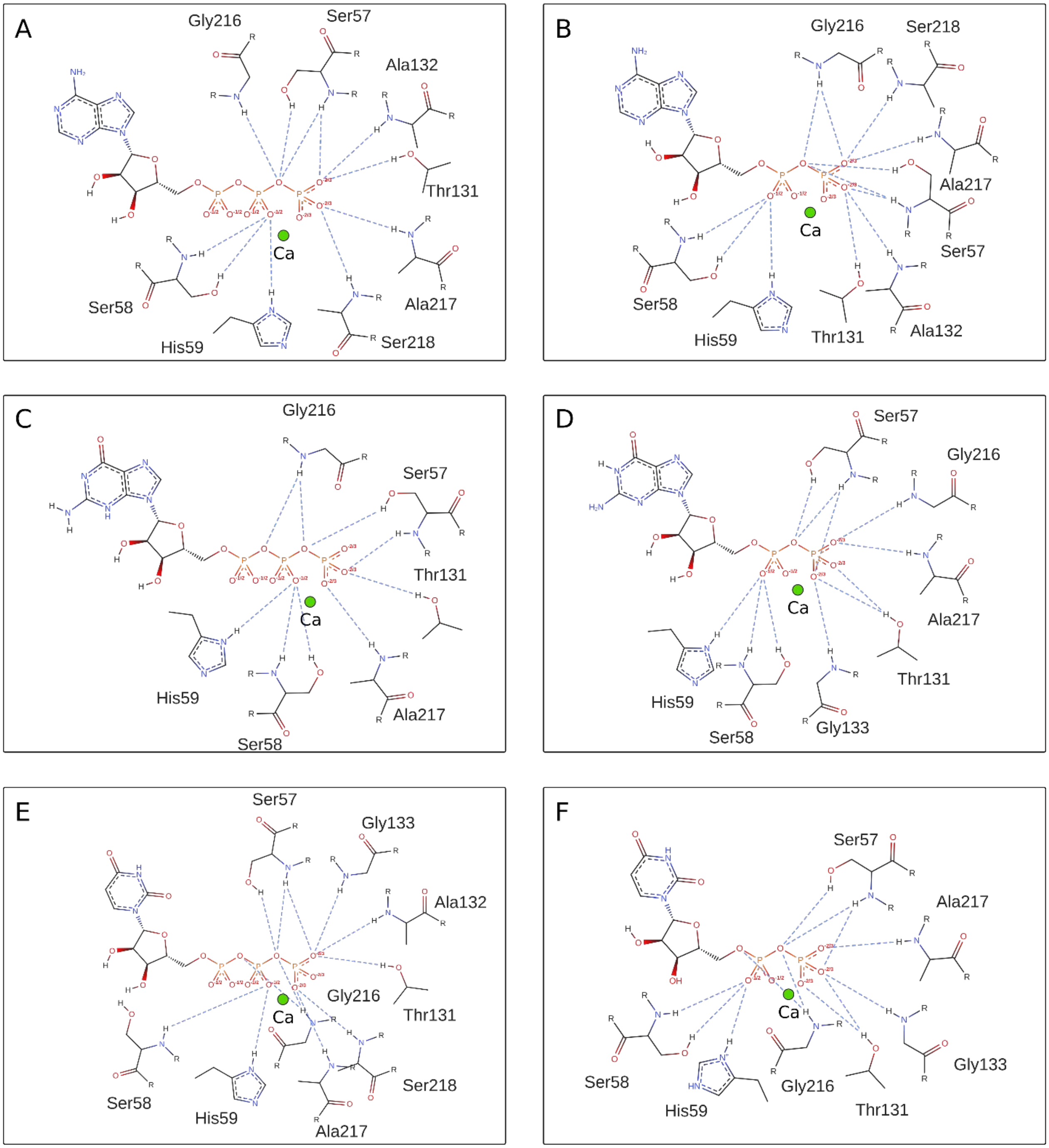

Figure S5 – 2D protein-substrate predicted interaction. A-F HsNTPDase1 interaction with ATP, ADP, GTP, GDP, UTP, UDP, respectively.

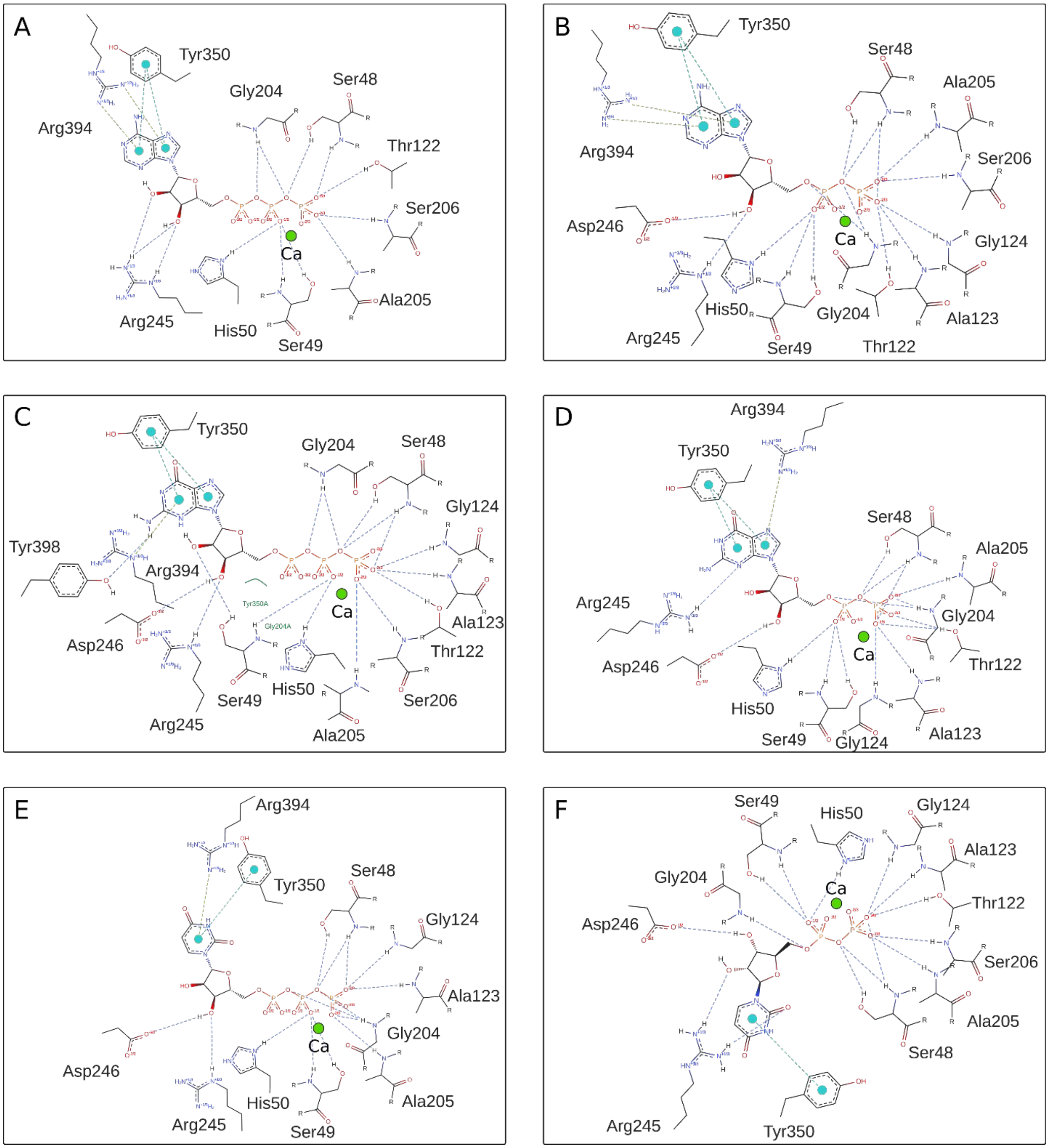

Figure S6 – 2D protein-substrate predicted interaction. A-F HsNTPDase2 interaction with ATP, ADP, GTP, GDP, UTP, UDP, respectively.

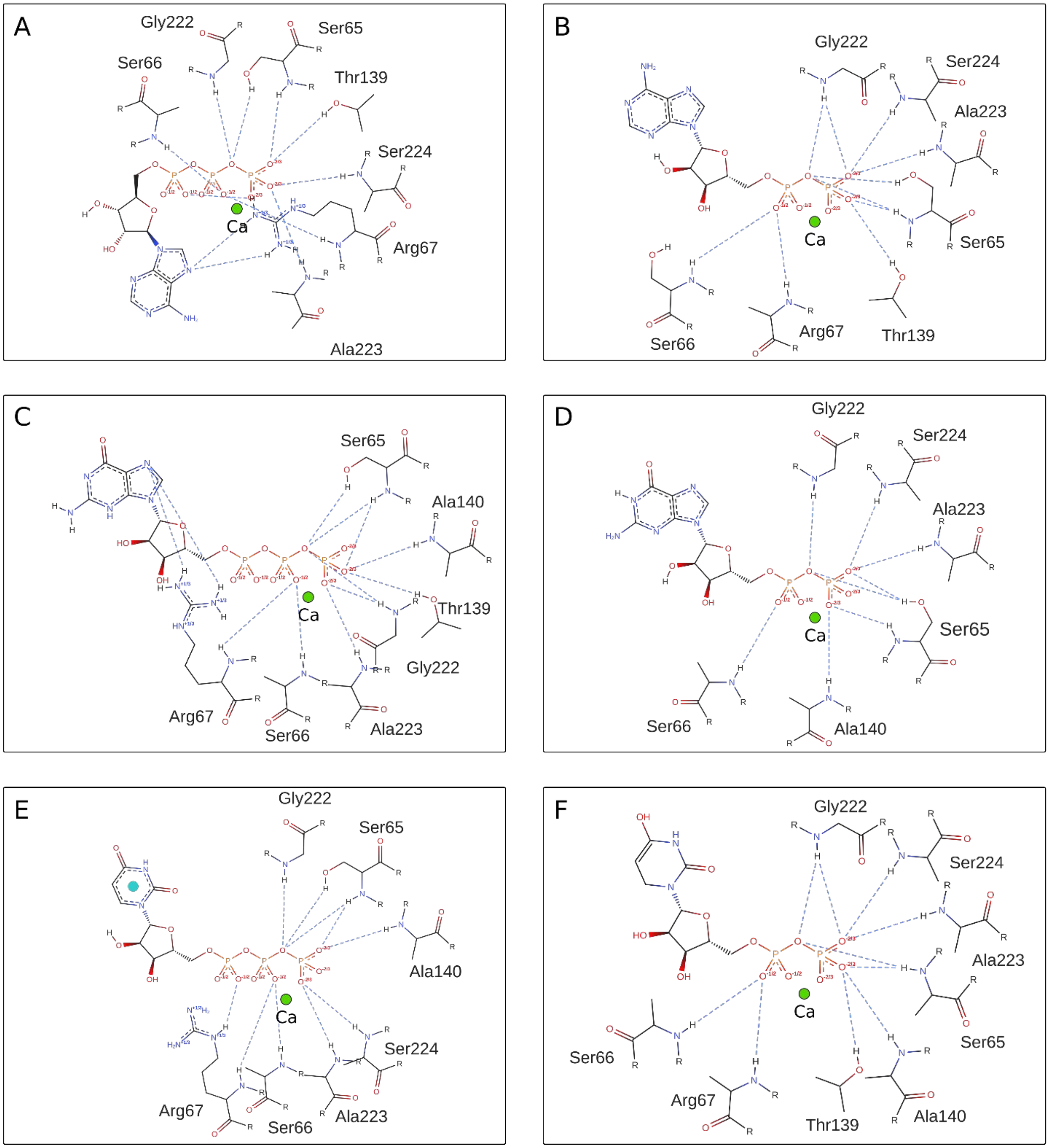

Figure S7 – 2D protein-substrate predicted interaction. A-F HsNTPDase3 interaction with ATP, ADP, GTP, GDP, UTP, UDP, respectively.

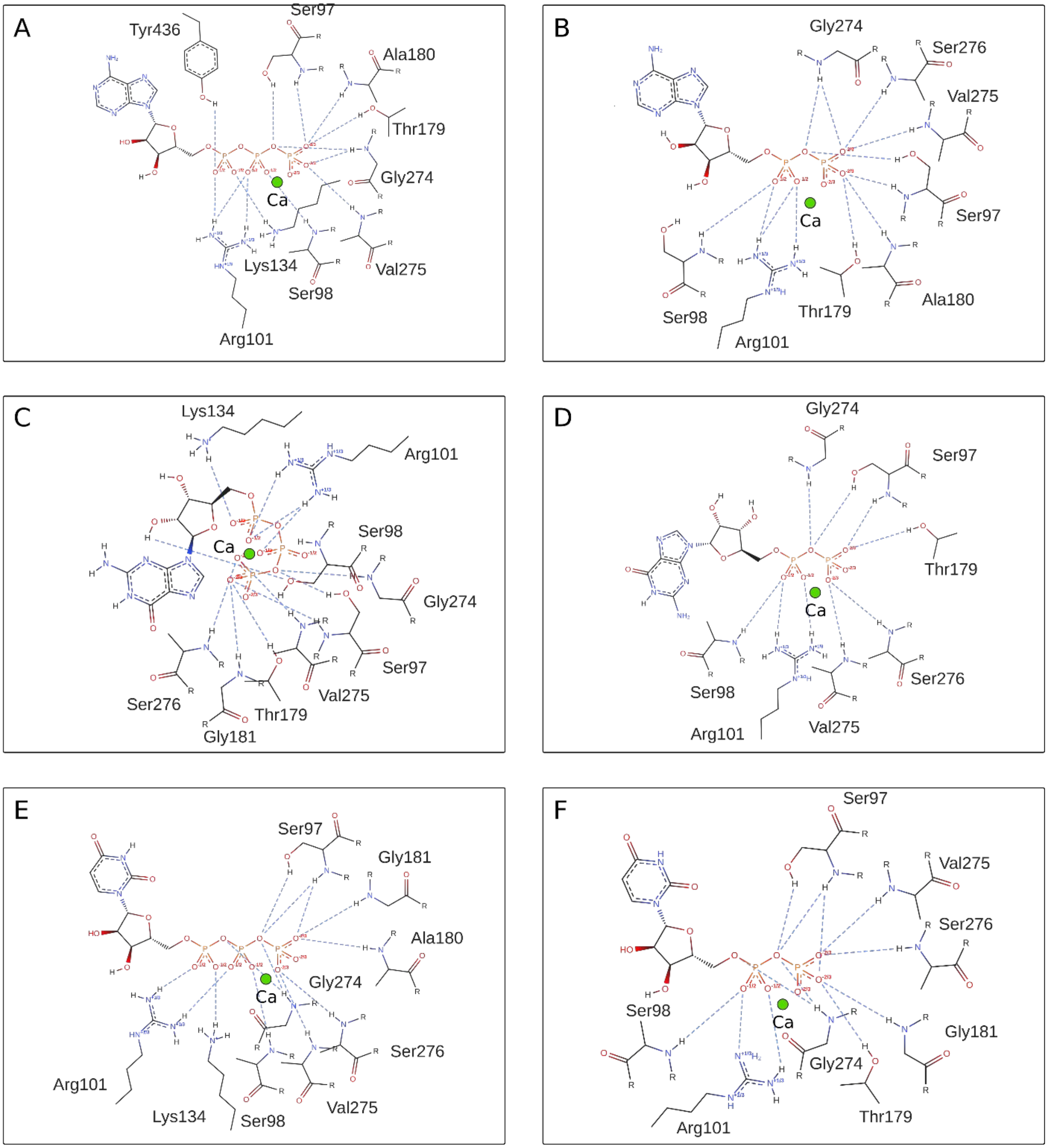

Figure S8 – 2D protein-substrate predicted interaction. A-F HsNTPDase4 interaction with ATP, ADP, GTP, GDP, UTP, UDP, respectively.

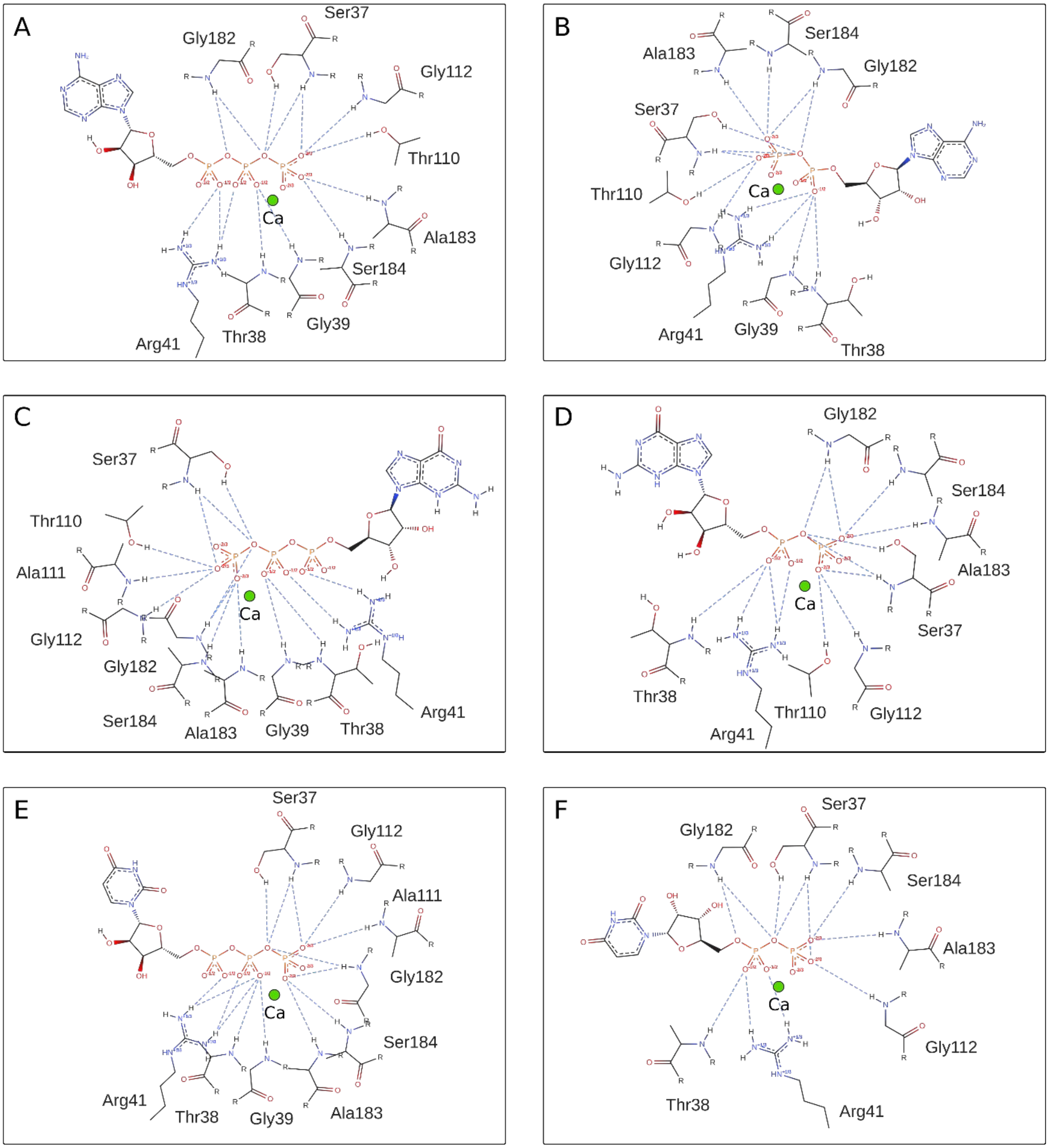

Figure S9 – 2D protein-substrate predicted interaction. A-F HsNTPDase5 interaction with ATP, ADP, GTP, GDP, UTP, UDP, respectively.

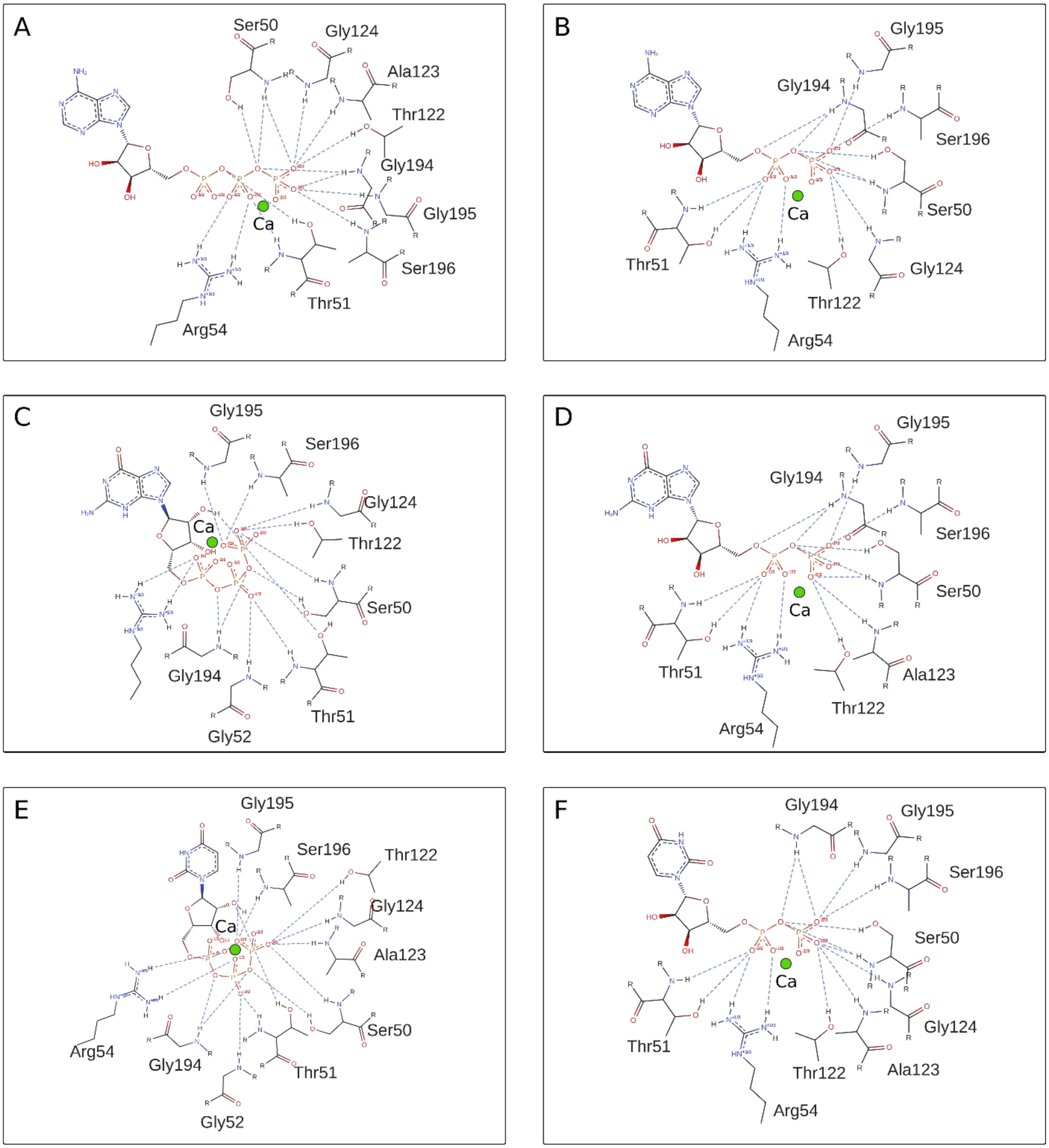

Figure S10 – 2D protein-substrate predicted interaction. A-F HsNTPDase6 interaction with ATP, ADP, GTP, GDP, UTP, UDP, respectively.

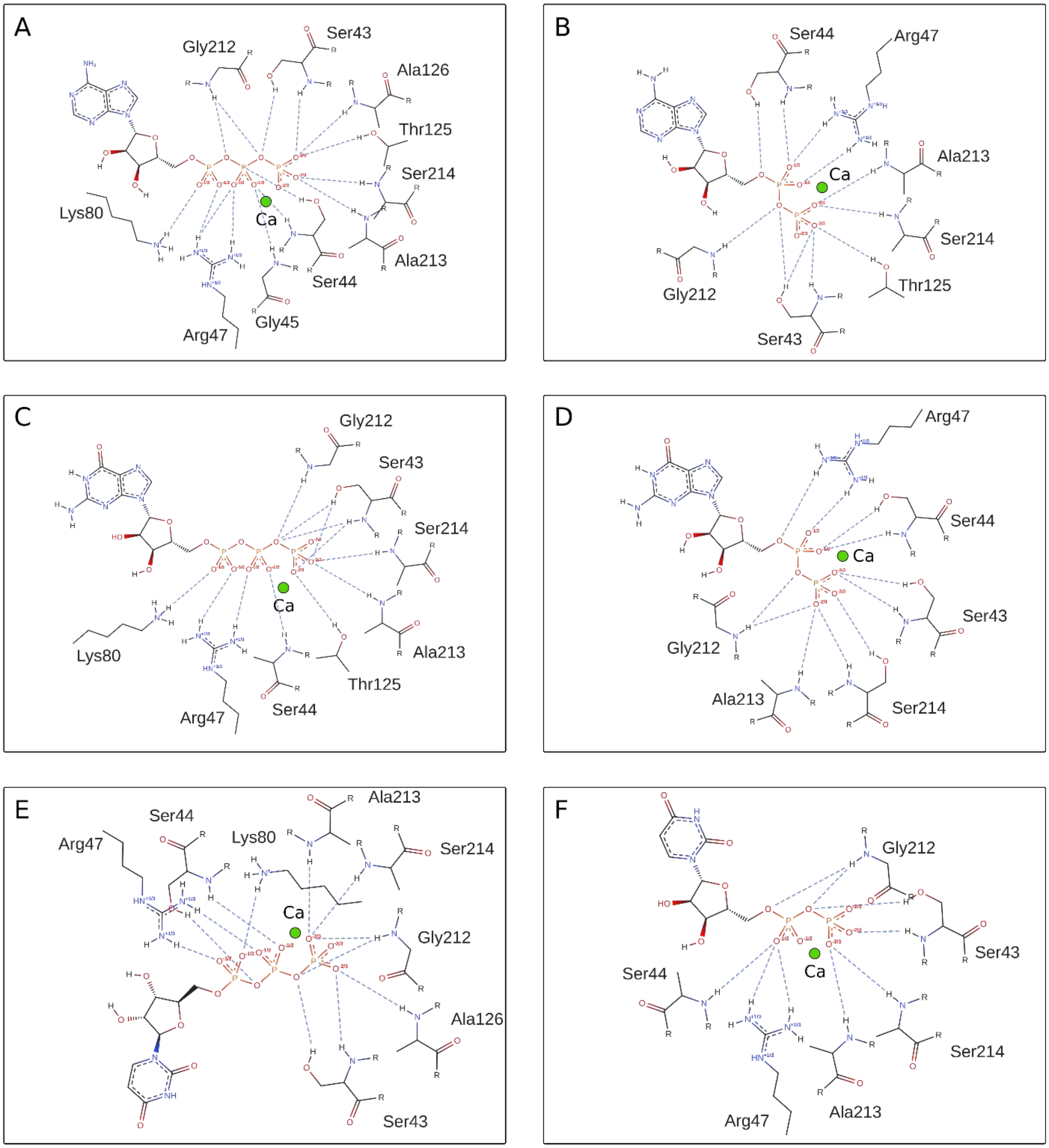

Figure S11 – 2D protein-substrate predicted interaction. A-F HsNTPDase7 interaction with ATP, ADP, GTP, GDP, UTP, UDP, respectively.

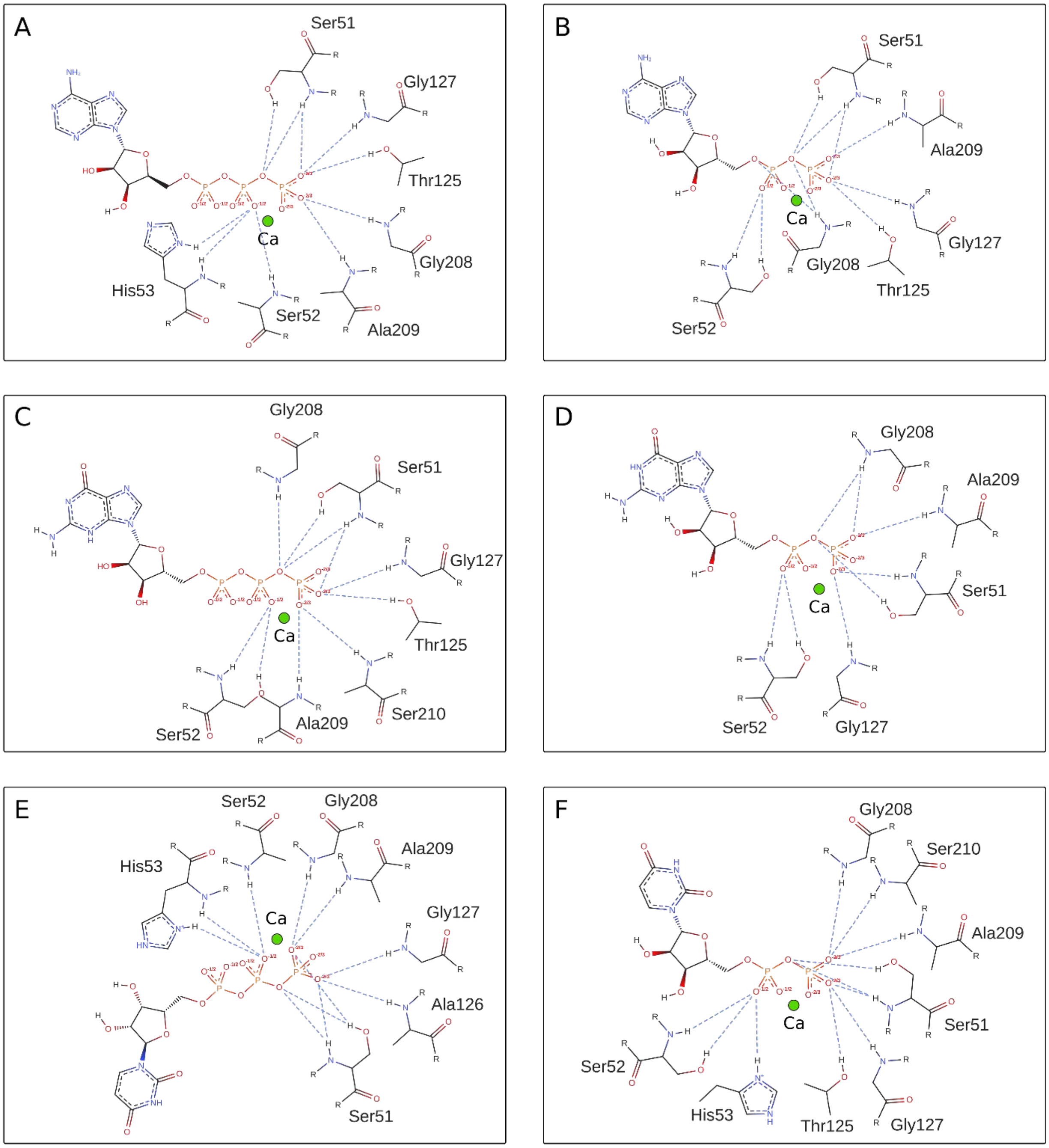

Figure S12 – 2D protein-substrate predicted interaction. A-F HsNTPDase8 interaction with ATP, ADP, GTP, GDP, UTP, UDP, respectively.

Table S1 – Modeled enzymes

Enzyme	UniProt Entry Code	Modeled residues
HsNTPDase1	P49961	46-467
HsNTPDase2	Q9Y5L3	37-452
HsNTPDase3	O75355	54-476
HsNTPDase4	Q9Y227	86-543
HsNTPDase5	O7536	26-408
HsNTPDase6	O75354	39-421
HsNTPDase7	Q9NQZ7	32-481
HsNTPDase8	Q5MY95	40-457

UniProt entry and regions of the sequence modeled for each HsNTPDase.

Table S2 - Experimental structures with a productive substrate binding mode

Enzyme / PDB code	Cofactor/Substrate Analog	Asp DXG ACR1 - Cofactor Distance (Å)	Asp DXG ACR2 - Cofactor Distance (Å)
RnNTPDase2 / 3CJA	Ca/AMPPNP	5.0	5.1
RnNTPDase2 / 4BQZ	Mg/GMPPNP	4.9	4.8
RnNTPDase2 / 4BR2	Ca/UMPPNP	5.0	5.0
LpNTPDase1 / 4BRA	Mg/AMPPNP	4.7	4.8
LpNTPDase1 / 4BRD	Mg/AMPPNP	4.8	4.7
LpNTPDase1 / 4BRG	Mg/GMPPNP	4.8	4.8
LpNTPDase1 / 4BRK	Mg/UMPPNP	4.7	4.8
TgNTPDase1 / 4KH4	Mg/AMPPNP	3.9	5.7
TgNTPDase3 / 4A5A	Mg/AMPPNP	4.8	4.9

RnNTPDase2 / 4BR0	Ca/AMPNP	4.9	5.1
LpNTPDase1 / 4BRC	Mg/AMPNP	4.8	4.7
LpNTPDase1 / 4BRI	Mg/UMPPNP	4.7	4.7
LpNTPDase1 / 4BRL	Mg/GMV	4.7	4.7
LpNTPDase1 / 4BRE	Mg/50T	4.7	4.8
TgNTPDase1 / 4KH5	Mg/AMPNP	4.0	6.5

AMPNP- Adenosine-5'-[(α,β)-imido]diphosphate, AMPPNP- Adenosine 5′-[β,γ-imido]triphosphate, GMPPNP- Guanosine 5′-[β,γ-imido]triphosphate, GMV-guanosine-5'-phosphovanadate, UMPPNP- uridine-5'-[(beta,gamma)-imido]triphosphate, 50T-adenosine-5'-phosphovanadate.

,

Table S3 – Measurements and interaction of the transferred substrate to HsNTPDases.

Enzyme	Substrate	RMSD (Å)	Asp DXG ACR1- Cofactor Distance	Asp DXG ACR4 - Cofactor Distance
NTPDase1	ATP	0.32	5.1	5.1
NTPDase1	ADP	0.26	4.8	6.0

NTPDase1	GTP	0.35	5.1	5.4
NTPDase1	GDP	0.32	4.9	5.2
NTPDase1	UTP	0.34	5.3	5.0
NTPDase1	UDP	0.94	4.9	5.1
NTPDase2	ATP	0.26	5.2	5.1
NTPDase2	ADP	0.25	5.7	4.9
NTPDase2	GTP	0.30	5.0	5.4
NTPDase2	GDP	0.13	5.8	5.0
NTPDase2	UTP	0.28	5.3	5.0
NTPDase2	UDP	0.87	5.6	4.9
NTPDase3	ATP	0.32	4.9	5.0
NTPDase3	ADP	0.38	5.1	5.1

NTPDase3	GTP	0.27	5.1	5.1
NTPDase3	GDP	0.38	5.5	4.6
NTPDase3	UTP	0.50	5.1	4.8
NTPDase3	UDP	0.92	5.7	4.5
NTPDase4	ATP	0.39	5.1	5.1
NTPDase4	ADP	0.63	5.4	4.9
NTPDase4	GTP	0.31	5.0	5.3
NTPDase4	GDP	071	5.0	5.4
NTPDase4	UTP	0.33	5.1	5.4
NTPDase4	UDP	0.95	5.5	5.0
NTPDase5	ATP	-	4.6	5.1
NTPDase5	ADP	0.35	4.7	4.8

NTPDase5	GTP	0.54	4.7	4.9
NTPDase5	GDP	0.33	4.9	4.9
NTPDase5	UTP	0.32	4.9	5.1
NTPDase5	UDP	0.81	5.0	5.0
NTPDase6	ATP	0.41	5.1	4.9
NTPDase6	ADP	0.30	5.1	5.3
NTPDase6	GTP	0.36	4.9	4.9
NTPDase6	GDP	0.37	4.9	4.7
NTPDase6	UTP	0.44	4.9	4.8
NTPDase6	UDP	0.91	4.7	5.6
NTPDase7	ATP	0.41	5.1	5.4
NTPDase7	ADP	0.64	5.1	5.0

NTPDase7	GTP	0.27	4.8	5.7
NTPDase7	GDP	0.62	5.0	5.4
NTPDase7	UTP	0.32	4.6	5.9
NTPDase7	UDP	0.89	4.9	5.7
NTPDase8	ATP	0.44	5.0	5.6
NTPDase8	ADP	0.38	5.4	4.8
NTPDase8	GTP	0.46	5.6	4.7
NTPDase8	GDP	0.19	4.6	5.1
NTPDase8	UTP	0.77	5.0	5.3
NTPDase8	UDP	0.79	5.4	5.0
